# Nanoparticles-based phototherapy systems for cancer treatment: Current status and clinical potential

**DOI:** 10.1016/j.bioactmat.2022.11.013

**Published:** 2022-12-05

**Authors:** Jiachen Li, Shiqi Wang, Flavia Fontana, Christos Tapeinos, Mohammad-Ali Shahbazi, Huijie Han, Hélder A. Santos

**Affiliations:** a Department of Biomedical Engineering, W.J. Kolff Institute for Biomedical Engineering and Materials Science, University Medical Center Groningen, University of Groningen, Ant. Deusinglaan 1, Groningen, 9713 AV, the Netherlands; b W.J. Kolff Institute for Biomedical Engineering and Materials Science, University Medical Center Groningen, University of Groningen, University of Groningen, Antonius Deusinglaan 1, 9713 AV, Groningen, the Netherlands; c Drug Research Program Division of Pharmaceutical Chemistry and Technology, Faculty of Pharmacy, University of Helsinki, Helsinki, FI-00014, Finland

**Keywords:** Phototherapy, Cancer, Nanoparticles, Therapeutic effect, Clinical potential

## Abstract

Remarkable progress in phototherapy has been made in recent decades, due to its non-invasiveness and instant therapeutic efficacy. In addition, with the rapid development of nanoscience and nanotechnology, phototherapy systems based on nanoparticles or nanocomposites also evolved as an emerging hotspot in nanomedicine research, especially in cancer. In this review, first we briefly introduce the history of phototherapy, and the mechanisms of phototherapy in cancer treatment. Then, we summarize the representative development over the past three to five years in nanoparticle-based phototherapy and highlight the design of the innovative nanoparticles thereof. Finally, we discuss the feasibility and the potential of the nanoparticle-based phototherapy systems in clinical anticancer therapeutic applications, aiming to predict future research directions in this field. Our review is a tutorial work, aiming at providing useful insights to researchers in the field of nanotechnology, nanoscience and cancer.

## Highlights

• The history and mechanisms of cancer phototherapy are introduced.
• Recent representative nanoparticle-based cancer phototherapy studies are reviewed.
• Innovative nanoparticles designs for the cancer photothermal and cancer photodynamic therapy are highlighted.
• Excellent therapeutic effect of the combinatory cancer therapy based on the cancer phototherapy nanosystems are highlighted.
• Feasibility and potential clinical applications of nanoparticle-based cancer phototherapy nanosystems.

## Graphical abstract

**Figure:**
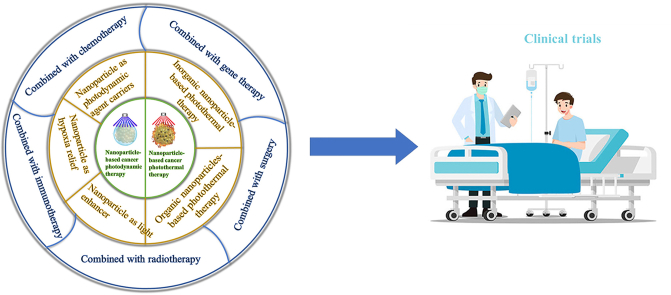

## 1 Introduction

### 1.1 The development of phototherapy

As early as three thousand years ago, sunlight-based phototherapy (PT) was recorded and carried out for a variety of diseases treatment, from rickets to many skin disorders [1,2]. It is considered to be related with the sun worship in human society at that time, i.e., believing the treatment effect was attributed to the red light and solar heat [3,4]. Although the human society has been moving forward after that, until the middle of the 19th century, heliotherapy was the only form of PT [1]. The attempts of the modern PT started with the advanced research development of optics, electricity and the invention of artificial light sources [1,4], such as the emergence of the “Sun Sanatoria” [5] and the combined PT with climatic treatment against tuberculosis epidemic [6]. The breakthrough was the published treatments on lupus vulgaris with the filtered sunlight or electric carbon arc torch-ultraviolet (UV) radiation, all conducted by Nils Ryberg Finsen (1860–1904) from 1893 to 1903. These treatment cases marked the establishment of the modern PT. Finson himself received the Noble Prize in Medicine in 1903 for his foundational work on modern PT [1,3]. After that, especially benefited from the arrival of the coherent and monochromatic laser by Theodore H. Maiman (1927–2007), modern PT utilizing various artificial light sources has been introduced into the research and treatment of other new categories of diseases in addition to those related to the skin [7]. Such early explorations include natural sunlight treatment on retinal disorders by Gerhard Meyer-Schwickerath (1920–1992) [8], allowing the present laser application in ophthalmology, and the clinical PT treatment on the neonatal jaundice with blue light in the range of 460–490 nm [9].

### 1.2 Cancer PT

Cancer has become the leading cause of death and the vital barrier to the life expectancy increase in every country of the world and will be the main cause of premature death (before 70 years old) globally in this century [10]. According to the statistics by the International Agency for Research on Cancer, in 2020, almost 10 million people died because of cancer (nearly one in six deaths). Notably, the estimated global cancer burden in 2040 is 28.4 million cases, with a 47% increase from 2020 [10], while the predicted cancer incidence in 2070 will double as in 2020 based on the current trend [11]. Modern PT has long been used for cancer treatment, just shortly after the PT trials on the retinal detachment in the 1960s [7]. In the early stages of cancer PT, laser was directly manipulated to thermally ablate the tumor tissue through its irradiation-induced heating [12,13], and the caused cancer cells damage can be considered as the results of cancer thermal therapy (TT) [14], which is considered as the predecessor of cancer photothermal therapy (PTT) [7]. But then the urgent limitations of the laser-based cancer TT were also unfolded, such as how to align the laser accurately on the malignant cancer cells to selectively heat the tumor site without significantly increasing the temperature of the surrounding normal tissues. The heating was also restricted by the laser penetration depth and the light-absorption by the endogenous substances, i.e., chromophores or water. The high-power density of the used laser brought issues about the treatment safety [7,15].

To overcome the hindrance occurred in the laser cancer TT and improve the cancer PT efficiency, exogenous and administered photosensitive agents-based cancer PT was established and has been developed until today [[16], [17], [18], [19]]. The photosensitive agents are expected to absorb the light source energy and then convert it into another form of energy for the therapeutic effect, which lowers the required power density of the light source.

Moreover, the targeting and selectivity of the treatment can be controlled and improved if the photosensitive agents also provide the imaging guidance. Depending on the functions of the agents and treatment routes, cancer PT mainly includes the PTT [20,21]and photodynamic therapy (PDT) [22,23].

#### 1.2.1 Cancer PTT

**Scheme 1:**
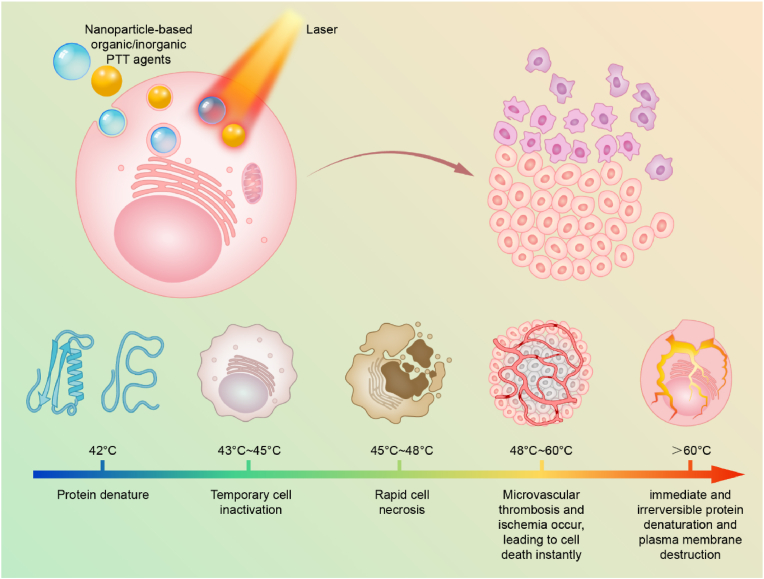
Schematic illustration of NPs-based cancer PTT.

In cancer PTT, the specific treatment modality is based on the heating of the cancer cells or the tumor tissue in local area, and the heating is mainly caused by the collisions between the light-excited PTT agents and the surrounding molecules in the tumor for the return to the ground state [7,24]. Generally, when the tissue temperature increases up to 42 °C, protein denaturation and temporary cells inactivation both occur. When it goes to 43–45 °C, long-term cells inactivation occur, leading to the local oxidative stress. When it goes to 45–48 °C, rapid cell necrosis happens. When it is at 48–60 °C, microvascular thrombosis and ischemia occur, leading to cells death instantly [25] (Scheme 1). With specific gold (Au) nanospheres as examples, more detailed physical analysis of PTT effect in cell and tissue level are discussed in the review by Qin et al. [26]. The main effect on cells and tissues after laser irradiation in PTT is the induction of a thermal injury whose kinetic follows the Arrhenius kinetic model, displaying an acceleration directly proportional to the temperature at which the biological sample is exposed [26]. On top of the thermal injury, microcavitation phenomena can mechanically injure the cells. The physiological effects of PTT in cancer cells are temperature-dependent [27]. Before temperature in the tumor increases up to 43 °C, it is ineffective towards cell killing, showing no effect on the percentage of apoptotic, necroptotic or necrotic cells *in vitro* despite the occurrence of cells temporary inactivation. On the other side of the spectrum, when irradiated tumor tissues reach temperature around 49 °C and higher, the prominent cell death path is necrosis, with more than 50% of the cells treated not responsive to inhibitors of apoptosis or necroptosis (caspase or RIPK1 inhibitors Z-VAD and Nec-1) [27]. Intermediate temperatures between 43 and 49 °C result in similar levels of apoptosis and necroptosis with only a limited necrosis. Furthermore, in case of shorter irradiation time and lower temperature reached, the process of apoptosis is initiated through the intrinsic pathway, regulated by Bcl-2 [28]. Recently, the interest in PTT-based cancer cell death has been focused on the type of cell death, aiming to induce immunogenic cell death able to activate the immune system [29]. The analysis of the differences in the gene expressions before and after irradiation of melanoma cells have identified mainly changes in the genes associated with the immune response, further supporting the case of a milder PTT treatment [30]. However, the thermal cell death promoted by PTT can also induce autophagy in the cancer cells, which require combination of PTT with anti-autophagy treatments to increase the efficacy of the therapy [31]. Combination therapies can also be useful to induce ferroptosis and to further increase the fraction of cells undergoing necroptosis [31] . Based on the principles of the cancer PTT, the ideal PTT agents should have following characteristics: strong light absorption capacity, excellent and stable light-to-heat conversion efficiency and brilliant biosafety and biocompatibility. A major PTT agent category comprises of organic molecules-based photosensitive agents [32], represented by indocyanine green (ICG). Besides, a large number of organic and inorganic nanoparticles (NPs) were developed as PTT agents or PTT enhancers [16,25,33,34], due to the unique transport, biological and optical properties [35,36].

Inorganic NPs mainly includes the metallic NPs represented by Au NPs and silver (Ag) NPs, carbon NPs represented by carbon nanotubes and graphene, quantum dots (QDs) represented by cadmium sulfide (CdS), rare earth doped NPs represented by trivalent neodymium (Nd^3+^)-based NPs and porous silicon NPs (PSiNPs). Organic NPs mainly include near-infrared (NIR)-dyes-delivery NPs and conductive polymers-based NPs represented by polypyrrole (PPy) NPs and polydopamine (PDA)-based NPs (Table 1). In the mid-1990s, the silica-Au nanoshells-based PTT has been utilized for the first clinical PTT trials, and finally commercialized in 2008, as AuroShell®Particles [37].

**Table 1:** Application of inorganic NPs in PTT and as PTT-based theranostic agents.

Inorganic NPs type	Size	ΔT °C	*In vitro* anti-cancer effect	Particles injection dosage of *in vivo* anti-cancer study	*In vivo* anti-cancer effect	Reference
Au nanorods (GNR)	55.1 nm length, 14.1 nm diameter	+25 °C in 10 min, max +64 °C with laser power 5W/cm^2^	60% cell viability reduction in MCF-7 cells	0.2 mg in Au	PTT alone is not effective in completely slowing xenografted MCF-7 tumor growth. Combination PTT/PDT effectively eradicates tumors	[98]
Gadolinium oxide-coated GNR	12 × 50 nm	+29 °C after 3 min exposure to 1.5 W/cm^2^; PTT transduction efficiency ∼56%	*N/A*	0.5 mL (no concentration information)	Dynamic fluorescence imaging of the real time procedure; 19.5 °C increase in the temperature in 5 min (0.55 W/cm^2^); formation of large necrotic area in implanted CC-531 rat colon adenocarcinoma tumor	[99]
Au NPs	5 nm before furin-induced aggregation; 103 nm after aggregation	ΔT max for aggregated particles 29.4 °C	Dose-dependent cytotoxicity in MDA-MB-68 cells after aggregation and laser irradiation	0.2 mg	Hyperthermia (*ca.* 44 °C); xenografted MDA-MB-468 human breast adenocarcinoma tumor rejection	[100]
Au NPs grown *in situ* on PSiNPs	*N/A*	ΔT max 38 °C for max laser power (2.6 W/cm^2^)	Almost 100% cell death on 4T1 cells	50 μL (1 mg/mL)	PTT always combined to vaccine or immune checkpoint inhibitor. Reduction in 4T1 tumor growth in primary tumor. Distal 4T1 tumors were eradicated.	[96]
MOF in gel (Polyoxometalates)	3.4 nm, incorporated in gel	ΔT max +35 °C (0.8 W/cm^2^)	95% reduction in cell viability of M21 cells.	100 μL (MOF: 0.3 mg)	Control over B16.OVA tumor growth in syngeneic mouse melanoma model. Hyperthermia (50 °C) and necrosis observed	[97]
MOF	32.3 nm short axis, 93.4 nm long axis	+30 °C	80% reduction in cell viability in HeLa cells	0.6 mg	S180 tumor model. PTT alone is not effective on tumor growth. Combination of PTT and RT reduces tumor growth	[101]
Carbon nanodots	8–20 nm	ΔT max +43.6 (2 W/cm^2^) with concentration of 200 μg/mL	*N/A*	0.1 mg	PAI of the H22 xenografted tumor and tumor targeting after intravenous administration. Intra tumoral administration needed for highest antitumor efficacy	[102]
Carbon nanodots	4.7 nm, 20 nm after intracellular aggregation	∼42% photothermal conversion efficiency	82% cell death in HepG2 cells	*N/A*	*N/A*	[103]
Carbon nanotubes (single wall)	*N/A*	ΔT max 43 °C (1.5 W/cm^2^)	60–70% reduction in cell viability in different pancreatic cancer cell lines	0.06 mg	BXPC orthotopic pancreatic tumor accumulation and optically guided PTT. Hyperthermia (+50 °C) and necrosis.	[104]
Cu selenide	6–13 nm depending on reaction conditions	Shift in absorbance towards NIR window II by increasing the size of the particles. Photothermal conversion efficiency: ∼42%, ∼36%, ∼19% for 808, 980 and 1210 nm laser	*N/A*	*N/A*	*N/A*	[105]
Ag sulfide	5–40 nm depending on the reaction conditions	*ΔT* max +30 °C for highest particle concentration (10 mmol) or highest laser setting (2.5 W/cm^2^)	60% reduction in cell viability in 4T1 and MCF-7 cells after laser irradiation (2.0 mmol)	Low dose group: 25 μmol/kg High dose group: 50 μmol/kg	*In vivo* PAI and tumor accumulation; dose-dependent control over the tumor growth in 4T1 tumor model	[106]

#### 1.2.2 Cancer PDT

**Scheme 2:**
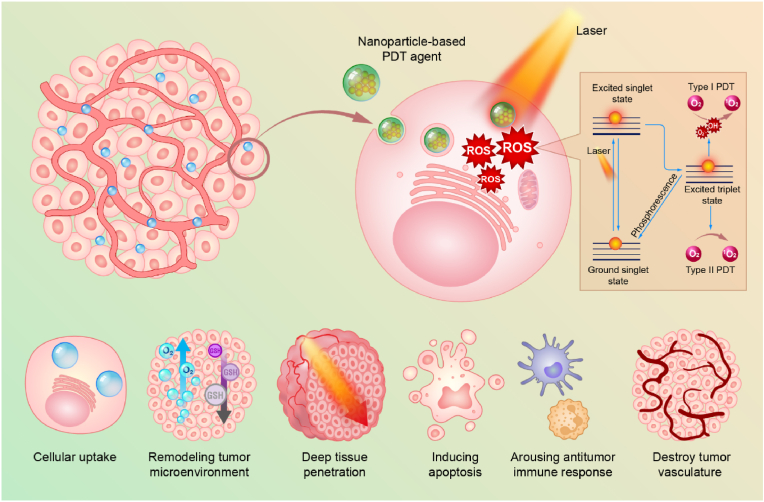
Schematic illustration of NPs-based cancer PDT.

In cancer PDT, photosensitive agents are referred as photosensitizers (PSs), and unlike the PTT agents, the specific treatment modality is based on the reactive oxygen species (ROS) generated by the PSs after being excited by the light source [38,39]. While being irradiated, after absorbing the energy from the photon, most of the current PSs transfer into a long-lived excited state, called triplet state [40,41]. Subsequently, the ROS are directly produced through two mechanisms: Type I and Type II (Scheme 2). In Type I, the PSs react with substrates, e.g., the cells membranes or some intracellular molecules, transferring a hydrogen atom to them to form radicals or radical ions. Then the formed radicals continues the reaction with oxygen to generate the oxygenated products, such as highly reactive singlet oxygen (^1^O_2_) [23]. In Type II, the triplet state of the PSs can directly transfer its energy to oxygen, forming ^1^O_2._ Besides the two predominate mechanisms, ROS also can be generated in other ways. After being irradiated, in the semiconductor NPs, the ROS can be produced from the reaction between the O_2_ (or H_2_O) and the separated electrons and holes from the generated electron-hole pairs [42,43]. In addition, it has been reported that ROS can be produced during the laser-induced potent heating on the metal NPs, which is either because of the reactions between the energetic hot-electrons and the surrounding media or the electrons moving over the barrier and reaching the surrounding media after they absorb enough energy [[44], [45], [46]]. Besides, during the Polyoxometalate-based radiotherapy (RT) process, the ROS can be produced due to the generated Auger and Compton electrons reacting with surrounding oxygen or water [47]. The readers are referred to Nosaka et al. for the detailed of the ROS generation mechanisms [48]. Accompanied by the generated ROS, PDT destructs the tumor tissues mainly by three mechanisms [39,49,50]: directly killing the cancer cells, inducing the vascular damage, and activating the immune response against tumor (Scheme 2). In addition, it also has been reported that the ROS, which was generated by the irradiated Au NPs also can cause the deoxyribonucleic acid (DNA) damage [24].

According to the ROS generation mechanisms discussed above, PSs, oxygen and light all play critical roles in the effective ROS generation and PDT outcomes, and the PSs is the essential one. The ideal PSs should have strong light absorption capacity, excellent and stable light-to-ROS conversion efficiency and brilliant biocompatibility. Most of the common PSs are organic molecules [42,51,52], and the representative categories are listed below. The first category is the small organic PSs, mainly including the most extensively studied porphyrins-based molecules and the commonly used indocyanine dyes-based molecules. The second representative category is the frequently reported aggregation-induced emission dyes (AIE)-based PSs. The third category is the noble metal complexes PSs, which are represented by Ruthenium (Ru)(II) complexes-based ones and Au(III) complexes-based ones. The fourth is organic frameworks compounds PSs, which mainly includes the metal-organic frameworks (MOFs) and covalent organic frameworks (COFs) [43]. The fifth representative category are the polymer-based PSs, which are represented by polyfluorene and semiconductor polymers. Besides these mentioned organic-based PSs, series of inorganic NPs has been developed and fabricated as the PDT PSs [42,53,54], such as the carbon NPs PSs, silicon NPs PSs, black phosphorous PSs and Titanium dioxide (TiO_2_) PSs [42,[53], [54], [55]]. The first PDT clinical trials on human were initiated by Kelly et al. in 1976 for bladder cancer therapy, with haematoporphyrin derivate (HPD) as the PSs and the results showed that the HPD slowed the tumor growth, and the occurrence of tumor necrosis was observed in PDT area. Then after almost 17 years’ clinical trials, HPD-based PSs, Porfimer sodium, was finally approved for bladder cancer in Canada [23,56] .

As an effective and approved cancer clinical treatment, cancer PT has unique characteristics and comparative advantages, such as non-invasiveness, controlled treatment process and relatively fewer side effects. In addition, with the continuous development of the research, the cancer PT agents with excellent photo-properties (*e.g.*, broad absorption cross section or, low phototoxicity) have been reported elsewhere [16,32,42,[57], [58], [59]]. However, there are still obvious limitations in many other reported research, *e.g.*, the inadequate targeting ability of the PT agents, the insufficient light penetration and the possible phototoxicity issues, which pose challenges for further clinical development of PT.

Many good review articles on NPs-based cancer PT have been published so far elsewhere [53,60,61], and these reviews have different emphasis like focus on the NPs specific for PDT [[62], [63], [64]], specific NPs for PDT and PTT [65,66], or the clinical potential [7].

In this review, we summarize the development of cancer PT and recent representative research in NPs-based cancer PT to clarify the necessity, importance and feasibility of the NPs in cancer PT at this stage, not focus on the specific NPs or particular phototherapy type. First, the typical NPs-based PTT and NPs-based PDT nanosystems will be introduced to illuminate how NPs can enhance the efficiency of the cancer PTT and cancer PDT. Specifically, we focus on how smart design of NPs overcomes the inherent shortcomings of cancer PT. Then, the distinctive nanosystems with combined therapeutic efficacy are introduced to enlarge the scope of cancer PT when combined with other common cancer therapies (e.g., chemotherapy, immunotherapy, and RT). We focus on the synergistic effects from combined therapies, and how NPs cooperate the joint efforts to maximize the overall therapeutic outcomes. Finally, we introduce the current clinical trials of NPs-based cancer PT, followed by the discussion of the clinical potential and future direction of NPs-based cancer PT.

## 2 NPs-based PT for cancer treatment: current status

### 2.1 NPs-based PTT for cancer treatment

There are two basic approaches currently being adopted. One is developing NPs themselves into a PTT agent, and the other is utilizing the NPs to deliver PTT agents. Compared with the direct laser irradiation on the tissues or systemic administration of the organic PTT agents (e.g., ICG molecules), NPs-based PTT is characterized by multiple advantages, including the increased accumulation in the tissues of interests *via* passive or active targeting, and the tailored spatial-temporal control on the photothermal effect *via* engineering of the NPs. These advantages have greatly improved the local treatment efficacy and reduced the amount of the administrated photothermal agents, thus reducing the local and systemic side effects [67]. Furthermore, nanomaterials are characterized by high photothermal conversion efficiency resulting from the mesoscopic nature [68]. The small size and structure characteristics of NPs may result in localized surface plasmonic resonance (LSPR), followed by heat generation.

Historically, inorganic NPs, particularly Au NPs, have been the first type of NPs investigated in PTT *in vitro* [69,70]: the synthesis of Au NPs can be easily tuned to achieve structures characterized by different size and shape, with a control over the adsorption wavelength and the LSPR [70]. Nevertheless, Au nanosystems do not display optimal properties as PTT agents: the adsorption wavelength is often outside the optimal NIR window (the second NIR windows, 1000–1700 nm [71]). The photothermal conversion efficiency is not optimal and repeated administrations may induce accumulation and toxicity [72]. Thereby, recent studies on PTT agents are investigating alternative inorganic materials, such as carbon-based or semiconductors, focusing in particular on shifting the adsorption window to the second NIR window with higher light penetration depth [73], where the interference from water in tissues is minimized and a lower laser power can be employed [[74], [75], [76]]. Regarding photothermal conversion efficiencies, the organic-based PTT agents are characterized by similar to or slightly higher efficiency than Au NPs. Moreover, they can be degraded within the body, lowering the long-term toxicity, which have been shown in several examples of semiconducting polymeric NPs [[77], [78], [79]]. The fine-tuning of the particles’ characteristics, together with the use of regulatory approved materials, enabled in-depth investigation of the variables influencing the photothermal effect, as well as the initial translation from bench to bedside. In the progress of nanotechnology for PTT of cancer, as described in Section 1.2.1, a single-material based therapeutic nanosystem, organic or inorganic, is the first and still the most researched to date [68].

In this section, we introduce cutting-edge development of inorganic and organic NPs for PTT. Here, we focus on the NPs design, photothermal effect and PTT therapeutic effect. The physical mechanisms responsible for the transformation of light to heat will be briefly reviewed, as well as the imaging possibilities provided. NPs used in PDT will be introduced in Section 2.2, while NPs presenting both PTT and PDT effect will be described in Section 3.

#### 2.1.1 Inorganic NPs-based PTT

Inorganic nanomaterials display optimal characteristics for an efficient conversion of light into heat, requiring lower energy input compared with irradiation alone to achieve the same temperature in the tissue [80]. These nanomaterials can be tailored to achieve high photothermal conversion efficiency (e.g., ∼49% in carbon polyhedras doped with copper (Cu) NPs with lower power intensity needed, improving the biocompatibility of the treatment). The field is moving towards complexes and nanostructures of ultrasmall inorganic particles to facilitate the excretion of the particles, lowering the systemic toxicity. The three main classes of inorganic NPs used in PTT include noble metal, semiconductor/transition metal and carbon-based materials.

Irradiated noble metal and semiconductor/transition metal NPs can produce heat *via* LSPR. LSPR is the result of the confinement of a surface plasmon in a NPs with the size comparable to or smaller than the wavelength of light which is the plasmon excitation [81]. Thus, it is not present in bulk material or at the level of the atoms and can be originated by the oscillation of the electrons in the conduction band in response to an electromagnetic stimulus, such as the laser irradiation [82]. Upon the irradiation, if the collective electron oscillation in the NPs has the same frequency of the photons, the absorption band forms and its resulting photothermal effect appears [82]. The photothermal effect is achieved with the decaying of the high energy state oscillation which releases energy in the surrounding solution without further radiation [80]. The readers are referred to Sharma et al. for the detailed mathematical analysis of the LSPR effect [80].

Unlike the noble metal and semiconductor/transition metal NPs, the photothermal effect in carbon-based materials is achieved *via* adsorption of energy, conversion in vibrations of the C–C bonds in the reticule and relaxation with release of heat [80,83,84]. For carbon-based materials, the size and orientation of the material also influence the adsorption wavelength [83,85]. In the case of nanotubes, the fine tuning of the photothermal properties involves evaluating the diameter, length and number of walls [85]. The readers are referred to Jaque et al. for the detailed review about PTT mechanism of carbon-based nanosystems [25].

The NIR irradiation of NPs characterized by LSPR can also generate photoacoustic (PA) signal, which can be exploited for the imaging of the tumor [86]. The increase in the local temperature determined by the laser irradiation of the NPs results into an increase in the pressure, according to Equation (1):

(1) $$p_{0}=\beta ∙\frac{\Delta T}{\kappa }$$

where, β is the coefficient of the thermal expansion, and κ is the isothermal compressibility [87]. The waves of pressure induced by laser irradiation are collected with an ultrasound transducer and processed with software to generate PA images. Metal NPs are good contrast agents for photoacoustic imaging (PAI) because they do not suffer from photobleaching; however, a prolonged laser irradiation may induce a change in their morphology, decreasing the overall signal and the contrast compared to the background [87]. The size and shape of the nanosystems play a role in PA signal intensity recorded [88]. However, a general model correlating the dimensions with the photothermal and PA signal is still missing, preventing the rational development of NPs-aided PAI of tumors. Semiconductor/transition metal nanosystems such as Cu selenide can be advantageous compared to noble metal particles because, based on the production methods and eventual doping of the structures, they can shift the adsorption peak to the second NIR window at wavelengths between 1000 and 1700 nm where the background interference signal is mainly given by water and not from the tissue [89]. However, a disadvantage of PAI is the limited penetration depth (∼5 cm), which requires the development of probes both for the laser and for the transducer when the tissue of interest is not on the surface of the body [86,87].

Recent research in inorganic photothermal systems has focused on improving their biocompatibility by combining ultrasmall particles in nanoarchitectures and by increasing the photothermal conversion factor [65,90,91]. Ultrasmall particles (<8 nm) can be readily excreted by the kidneys, improving the biocompatibility of the system [92]. However, their size is too small to achieve LSPR in the NIR region and with a high photoconversion efficiency, and their renal excretion is too fast to achieve significant tumor accumulation [90]. The loading of multiple ultrasmall particles within the same nanoarchitecture finetunes the adsorption wavelength and the final PTT and PA effect [93].

Cassano et al. developed a passion-fruit like nanoassembly where ultrasmall Au NPs are embedded within a polymeric matrix further coated with a silica shell [94]. The irradiation at 808 nm of the obtained particles in aqueous solution determined an increase in the temperature of up to 58 °C for the maximum power setup (2.6 W), with a laser power of 1.1 W needed for the particles to exceed hyperthermia temperature in 250 s [95]. These structures can withstand repeated cycles of irradiation without damage or re-shaping of the nanoassemblies, maintaining the photothermal efficacy and a size suitable for renal excretion.

**Fig. 1:**
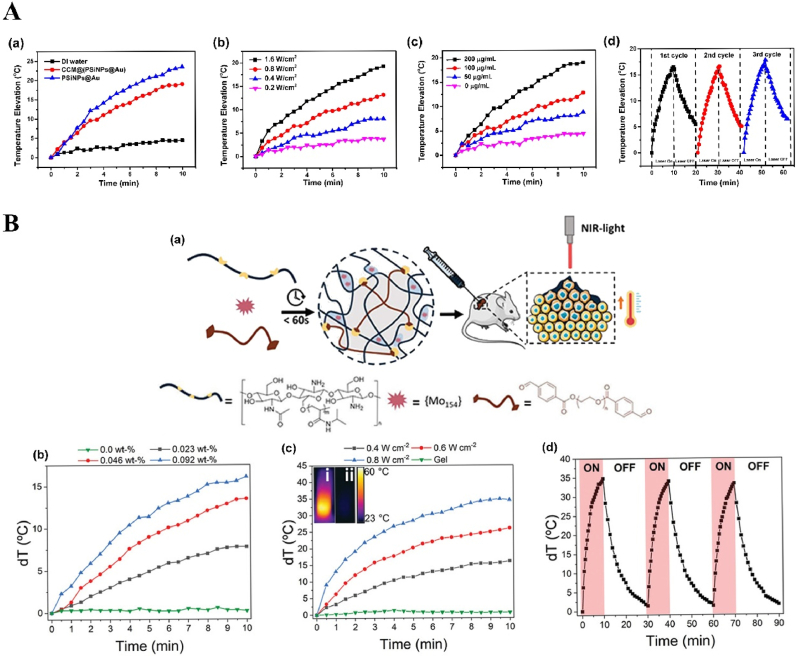
**Inorganic NPs-based innovative cancer PTT nanosystems.** **A. Photothermal profile of Au NPs grown in situ on PSiNPs**: (a) *ΔT* °C elevation of solution containing 200 μg/mL PSiNPs@Au NPs before and after coating with cancer cell membrane upon exposure to 808 nm laser at 1.6 W for 10 min; (b) Photothermal conversion of PSiNPs@Au NPs (200 μg/mL) after 10 min irradiation at different laser powers; (c) Photothermal conversion curve of PSiNPs@Au NPs at different concentrations irradiated for 10 min with laser intensity 1.6 W; (d) Photothermal stability upon repeated cycles of irradiation (10 min on, 10 min off). Adapted from Ref. [96]. Copyright *©* 2022, Wiley-VCH. **B. Photothermal profile of Mo**_**154**_**-based cluster**: (a) General scheme showing the design, preparation, and synergistic effects of PTT; (b) Photothermal conversion curve of MOF-gel at different concentrations of MOF after irradiation with a 808 nm laser for 10 min at 0.4 W; (c) Photothermal conversion curve of MOF-gel at the concentration of 0.92wt-% of MOF after 10 min irradiation at different laser powers (inset showing thermal images of i MOF-gel and ii gel after 10 min irradiation with 0.8 W laser power); (d) Stability of the photothermal effect upon repeated cycles of irradiation (0.8 W, 10 min) and 20 min of relaxation. Adapted from Ref. [97]. Copyright *©* 2021, Wiley-VCH.

Recently, Li et al. reported that Au NPs were grown *in situ* on PSiNPs: the obtained Au NPs were characterized by diameters between 15 nm and 40 nm as observed in transmission electron microscopy. This system presented both good photothermal properties and stability, with a *ΔT* of 20 °C upon irradiation with the 808 nm laser at 1.6 W and stability of the photothermal effect after three cycles of irradiation (Fig. 1A). The biocompatibility tested *in vitro* in 4T1 cells was optimal, with 90% of cells alive after 24 h of incubation with the system. The viability sharply decreased almost to zero when the wells containing cells and particles were exposed to a 1.6 W laser for 10 min. The photothermal efficacy *in vivo* was confirmed in a 4T1 subcutaneous tumor model: after a single administration of the nanosystem and a single PTT cycle, the tumor growth in the primary tumor was inhibited up to 20 days post treatment in most of the animals, while the distal tumors did not grow in 25 days due to the PTT-activated immune response against cancer. The animals treated with the NPs and PTT survived up to 60 days after tumor inoculation. Importantly, despite having sizes incompatible with renal excretion mechanisms, 4 repeated subcutaneous injections of the nanosystem did not cause any systemic toxicity as evaluated by hematoxylin and eosin (H&E) staining as well as by the animals’ body weight [96].

Guedes et al. also reported another NPs for cancer PTT, the Mo_154_-based cluster, composed of an early transition metal and oxygen, characterized by intervalence charge transfer transitions, which provide high photothermal conversion efficiency of ∼31% with a laser power of 0.8 W, higher than the conventional noble metal particles (13–21%). Furthermore, the MOF can withstand repeated cycles of laser irradiation maintaining the same efficiency (Fig. 1B). However, the MOF is toxic both in melanoma cells and in primary fibroblasts upon prolonged exposure. The loading of the MOF within a hydrogel prevents the toxic effect of free MOF. This injectable hydrogel was co-loaded with doxorubicin (DOX) as model drug and evaluated for efficacy in murine melanoma models: the tumor irradiation with a NIR laser at 808 nm resulted in an increase in the tumor temperature to 50 °C with induction of necrosis, significantly more than the temperature recorded in a tumor injected only with the vehicle. The laser irradiation and the pH- and laser-dependent release of DOX showed a synergistic effect on the tumor growth, improving the efficacy of either treatment alone [97].

Besides the examples discussed above, we include more recently published representative applications of inorganic NPs in PTT and as theranostic agents in Table 1, presented in terms of their size, photothermal performance, *in vitro* and *in vivo* therapeutic effects.

#### 2.1.2 Organic NPs-based PTT

The research into alternative organic NPs for theranostic cancer applications based on PTT flourished in the quest to develop biocompatible and biodegradable photothermal agents with similar photothermal conversion efficiency but lower toxicity compared to conventional inorganic NPs [107]. Organic-based materials display photothermal and PA effects following the non-radioactive relaxation of the high energy state induced by the photon adsorption during the laser irradiation [108]. We refer the readers to the review from Ng et al. for a complete description of the physical mechanisms of PTT in organic molecules [108] and we refer the readers to Zhen et al. for the comprehensive review on the engineering of semiconducting polymer-based NPs to increase their PA and photothermal efficiency [109]. The families of organic molecules investigated as PTT agents to be formulated in NPs include: cyanines, with ICG being the most studied, diketopyrroles, croconaines, porphyrins, polyaniline and PPy, dopamine and melanin [107]. Finally, semiconducting polymer-based NPs represent a class of organic polymeric NPs with photothermal conversion efficiency higher than inorganic materials such as Au or carbon nanotubes and biodegradability provided by careful engineering of the polymeric backbone's bonds [109,110].

Cyanine-based molecules are formulated in conventional NPs, including liposomes, micelles, poly lactic-co-glycolic acid particles, or human serum albumin-based systems [[111], [112], [113], [114]]. The NPs formulations aim to improve tumor accumulation, half-lives, photothermal effect and PAI capabilities of these molecules. For example, croconaine-loaded NPs, which were reported by Li et al., have shown high contrast in PAI, enabling a real time monitoring of NPs accumulation within the tumor [115]. Furthermore, the authors used croconaines with different adsorption wavelength to compare the accumulation of targeted and untargeted NPs; the irradiation of the particles with the 808 nm laser set at 1.0 W determined a photothermal conversion of 30 °C, comparable to Au nanosystems. Croconaines can also be modified to shift the adsorption in the second NIR window, enhancing the sensitivity, resolution and penetration of photothermal and PA effects [116].

Alternatively, PPy, PDA, porphyrins and melanin self-assemble into nanostructures with a tailorable adsorption in the two NIR windows and multiple imaging modalities. The photothermal conversion efficiency is similar to noble metal particles and can reach 30% for particles adsorbing in the first NIR window (808 nm) and up to 40% for particles adsorbing in the second NIR window (1064 nm). Huang et al. described mesoporous PDA NPs displaying the *ΔT* of 30 °C upon irradiation with 0.8 W/cm^2^ for 5 min at 808 nm. The system was encapsulated within cancer cell membrane to improve the targeting to the tumor tissue upon intravenous administration [117]. Semiconducting polymeric NPs are characterized by a photoactivable semiconducting polymeric core, which allows PTT, as well as PA or fluorescent imaging of tumors [118]. The main advantage of semiconducting polymeric NPs over other organic NPs for PTT is the presence of the photoactivator in the polymer, which is then precipitated into particles, compared to the loading of a molecule like indocyanine. Furthermore, the structure of the semiconducting polymeric NPs can be easily engineered in the backbone to introduce donor-acceptors to shift the absorption from NIR I to NIR II window, with enhanced efficacy *vs.* lower fluence of the laser and higher safety of the treatment [109,118]. Duan et al. recently reported a modification of two isoindigo-based semiconducting polymeric NPs (identified as PBFT-DIID) to present a strong absorption between 700 and 1000 nm, correlated with a photothermal conversion efficiency of 70.6%, with a concentration dependent increase in the temperature up to 60° within 5 min of laser irradiation at 0.5 W/cm^2^, and stable photoactivation in 5 repeated irradiation cycles, correlated with a complete tumor eradication in 4T1 subcutaneous tumor model after 6 min exposure to 0.8 W/cm^2^ over the tumor area [119].

**Fig. 2:**
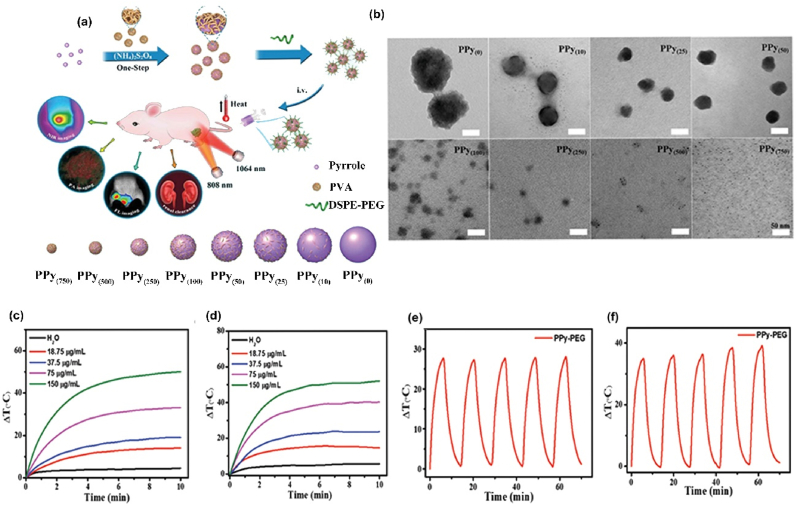
**Organic NP-based innovative cancer PTT nanosystems.** (a) Schematic presenting the synthesis pathway and the applications of the ultrasmall PPy-based NPs in PTT and imaging modalities; (b) TEM images displaying PPy NPs obtained after the addition of different amounts of poly vinyl alcohol (PVA); (c) Photothermal conversion curve of PPy-based NPs irradiated at 808 nm (1.0 W/cm^2^) for 10 min for different concentration of NPs; d) Photothermal conversion curve of PPy-based NPs irradiated at 1064 nm (1.0 W/cm^2^) for 10 min for different concentration of NPs; (e) Photothermal stability of ultrasmall PPy-based NPs after repeated cycles of irradiation at 808 (1.0 W/cm^2^); (f) Photothermal stability of ultrasmall PPy-based NPs after repeated cycles of irradiation at 1064 nm (1.0 W/cm^2^). Adapted from Ref. [120]. Copyright *©* 2021, Wiley-VCH.

Organic nanosystems present lower toxicity compared to the inorganic ones. However, in order to obtain the least side effects possible, organic NPs like PPy are formulated into ultrasmall particles excretable through the kidneys. For example, Zeng et al. reported ultrasmall PPy NPs with size suitable for renal excretion (2 nm), fluorescent, with high contrast in PA and the possibility to shift the adsorption to the second NIR window (Fig. 2(a and b)). These particles are characterized by high photoconversion efficiency (∼33% when irradiated with 808 nm laser and ∼42% when irradiated with 1064 nm laser) and photostability at both wavelengths (Fig. 2(c–f)) [120].

Other studies found the functionalization of diketopyrroles with fluorine and chalcogens (particularly selenium) is needed to increase the photothermal conversion efficiency from 32% to 62% due to π-π and F–H interactions [121]. The biocompatibility and biodegradability of semiconducting polymer-based NPs can be controlled *via* the type of bonds added to the polymeric backbone. Lyu et al. reported a biodegradable semiconducting polymer-based NPs with enhanced photothermal conversion efficiency and biodegradability by introducing vinylene bonds able to be enzymatically oxidized (*e.g.*, by myeloperoxidase in immune cells); the particles showed enhanced PA signal in a 4T1 tumor model, as well as tumor eradication after PTT treatment with a low laser intensity of 0.3 W/cm^2^ [78].

In addition to the examples discussed above, we include more recently published representative applications of organic NPs in PTT and as theranostic agents in Table 2, presented in terms of their size, photothermal performance, *in vitro* and *in vivo* therapeutic effects.

**Table 2:** Application of organic NPs in PTT and as PTT-based theranostic agents.

Organic NPs type	Size	Photothermal efficacy	In vitro anti-cancer effect	Particles injection dosage of *in vivo* anti-cancer study	In vivo anti-cancer effect	Reference
ICG loaded in silk fibroin NPs	165.9 nm	*ΔT* max 30 °C at the highest ICG concentration (20 ppm) laser power 1.5 W/cm^2^	Dose-dependent cytotoxicity in MCF-7 and HeLa cells, up to 90% toxicity for particles concentration of 20 μg/mL	No information was provided on volume or dose.	Control over xenografted MCF-7 tumor growth	[122]
ICG loaded in PLGA NPs	159 nm	ΔT max 20 °C, laser 1.0 W/cm^2^, ICG concentration 15 μM	90% reduction in cell viability in MCF-7 cells incubated for 24 h after laser irradiation	*N/A*	*N/A*	[111]
ICG conjugated to PEG pH sensitive micelles	30 nm at pH 7.4	ΔT max 34 °C for highest concentration of micelles. Higher PTT at acidic pH	100% toxicity towards A549 cells after laser irradiation (1 W/cm^2^, 5 min)	1.5 mg/kg (equivalent ICG)	Hyperthermia (55 °C, 1 W/cm^2^, 5 min), control over xenografted A549 tumor growth	[123]
Diketopyrrole derivative containing fluorine and selenium	60 nm (DLS), 50 nm (TEM)	Photothermal conversion efficiency 32% for unmodified particles, up to 62% for fluorine and selenide containing particles	Dose-dependent toxicity in A549 cells after laser irradiation	2 mg/kg	PAI of the tumor. Xenografted A549 tumors were eradicated.	[121]
PEG-PLGA NPs loaded with croconaines	180 nm	Photothermal conversion efficiencies between 32 and ∼35% in acidic solution (pH 6.5)	Dose-dependent reduction in cell viability (up to 90%) in MDA-MB-231 cells	5 mg/kg	*In vivo* multispectral PAI; antitumor efficacy in xenografted MDA-MB-231 tumor	[115]
Peptide-croconaines self-assembled NPs	20 nm at pH 7.4, 512 nm at pH 5.5	ΔT max 58 °C (1 W/cm^2^, 5 min)	Toxicity in Hu7 cells after laser irradiation	0.1 mL (2 mM)	*In vivo* NIR II imaging, PAI, hyperthermia (>40 °C), control over HepG2 tumor growth	[116]
Porphyrin-polymer NPs	133 nm	Photothermal conversion efficiency of 66%	Dose-dependent cytotoxicity in 4T1 cells after laser irradiation	0.04 mg	Hyperthermia (55 °C); control over 4T1 tumor growth	[124]
Porphyrin-diketopyrrole self-assembled particles	120 nm	Photothermal conversion efficiency of ∼63%	Dose-dependent cytotoxicity in HeLa cells with up to 70% reduction in cell viability	0.05 mg	Hyperthermia (60 °C); Eradication of tumor in xenografted HeLa tumors	[125]
Ultrasmall PPy NPs, PEG and PVA	2 nm, size dependent on PVA concentration	Photothermal conversion efficiency of ∼33% at 808 nm and ∼42% at 1064 nm	Dose-dependent cytotoxicity in U87 cells after irradiation with either 808 nm or 1064 nm laser	23 mg/kg	Fluorescence and PAI; hyperthermia (55 °C in 10 min, laser power 1.0 W/cm^2^); control over xenografted U87 tumor growth	[120]
Cancer cell membrane coated mesoporous poly dopamine NPs	250 nm	Photothermal conversion efficiency ∼39%	Dose-dependent and laser power-dependent toxicity in RM-1 cells	4 mg/kg for biodistribution study, no dose information for the PTT *in vivo* study	Hyperthermia (55 °C in 5 min); control over xenografted RM-1 tumor growth	[117]
Macrophage membrane coated poly dopamine NPs	159.6 nm	Photothermal conversion efficiency of 27%	Laser power-dependent cytotoxicity in 4T1 cells	10 mg/kg	Hyperthermia (*ca.* 50 °C); Control over 4T1 tumor growth and long term survival	[126]
Melanin NPs coated with a silica shell	100–150 nm	Photothermal conversion efficiency of 60% after silica coating, ∼67% for melanin particles before coating	Dose-dependent toxicity in 4T1 cells after laser irradiation (1064 nm, 1.0 W/cm^2^, 5 min)	0.1 mg	Control over tumor growth in 4T1 tumor model; hyperthermia (50 °C)	[127]
Semiconducting polymeric NPs two isoindigo modification	170 nm	ΔT°C max 27.3 °C (808 nm laser, 0.5 W/cm^2^, 7 min)	80% reduction in cell viability in 4T1 cells after incubation with 25 μg of particles followed by laser irradiation (808 nm, 1 W/cm^2^, 5 min)	0.05 mg	4T1 tumor eradication after laser irradiation (808 nm, 0.5 W/cm^2^, 6 min). Hyperthermia (65 °C)	[128]
Semiconducting polymeric NPs vinylene bonds in the backbone	36 nm	Photothermal conversion efficiency of 71 ± 2%	Dose- and laser power-dependent reduction in cell viability of 4T1 cells after irradiation for 8 min at 0.3 or 0.5 W/cm^2^	6 mg/kg	Hyperthermia (50 °C); Eradication of 4T1 tumor after irradiation for 6 min at 0.3 W/cm^2^	[78]

The undefined abbreviations in the table: polyethylene glycol (PEG), poly(lactide-co-glycolide) (PLGA).

### 2.2 NPs-based PDT for cancer treatment

As mentioned in Section 1, the PDT treatment is based on the generated ROS from light-activated PSs. Until now, PSs have been developed into three generations [60]. The first generation PSs were porphyrin-based PSs, which were developed in the 1970s and early 1980s and are represented by HPD. The second generation PSs are porphyrin-based porphyrinoid compounds or porphyrin-based macrocyclic structures, which were mostly developed since the late 1980s and are represented by chlorins. The third generation PSs are the various currently studied PSs, which aim to overcome the shortcomings of the former generations PSs [60].

For better PDT effects, the upgrade of existing traditional organic PSs has been investigated for long time, and efforts have been made to overcome the hypoxia limitation in the tumor microenvironment (TME) and the phototoxic effects from PSs. For example, recently, An et al. developed three organic PSs by biotinylating three typical PDT PSs, two fluorescein-derivatives and one protoporphyrin-derivative. These synthesized new biotinylated PSs with better ability of hypoxia tolerance could not only target the tumor, but also impressively enhance the production of the ROS even upon low-power white light irradiation (20–40 mW/cm^2^) by both Type I and Type II mechanisms. The generated ROS included both single oxygen and anion radicals. The results showed the generation of anion radicals *via* Type I mechanism was not susceptible to tumor hypoxia, compared with ^1^O_2_ generation *via* traditional Type II mechanism. This work provides a new strategy to design synergistic Type I/Type II PDT PSs to alleviate the tumor hypoxia [129]. The second example focuses on how to prevail over the undesirable phototoxic side effects caused by the slow metabolism of the PSs during the whole PDT process, which is one of the chief obstacles of the PDT clinical translation. Zhu et al. recently constructed a list of strongly fluorescent *seco*-chlorins with β-pyrrolic ring-opening structure (beidaphyrin (BP), beidapholactone (BPL)) and their zinc (II) derivatives (ZnBP and ZnBPL). The *in vitro* and *in vivo* experiments indicate that all the new developed PSs are featured with the ability of effective ROS generation, strong NIR absorption, and potent tumor PDT (82% tumor growth inhibition compared with control group). More importantly, experimental results showed that under the laser irradiation (700 nm, 200 mW/cm^2^), water soluble ZnBPL was converted to non-photocytotoxic, degradable and metabolizable beidaphodiacetamide (ZnBPD) by the generated O_2_^.-^, significantly relieving the possible phototoxic side effects [130].

Despite the progresses on the PSs, their limitations are still obvious, such as the easy photochemical bleaching, poor solubility, lack of lesion site targeting and the resulting systemic toxicity [131,132]. In addition to PSs, there are another two key factors closely affecting the therapeutic effect of the PSs-based PDT, i.e., the oxygen concentration during PDT and the light exposure during the excitation, such as the tissue penetration depth of the excitation light and light fluence rate (power per unit area of light given in watts per square meter, W/m^2^) [23]. Oxygen is the main source of the ROS, cancer cells killing tool, but the hypoxic TME poses significant obstacle for the PDT. Light is the energy source and the trigger of the whole PDT, and thus, the ultimate effective efficiency of the laser irradiation on the PSs also should be improved as much as possible [63].

In order to overcome these challenges and potentiate the possibility of PDT clinical applications, the NPs-based nanosystems are developed for cancer PDT. These nanosystems utilize the advantages of the NPs to enhance PDT, e.g., the large surface/volume ratios granting the drugs or PDT agents loading capacity, excellent surface modifiability endowing the conjugation of functional and targeting groups, and the preferable morphology of the NPs which may increase the uptake by the targeted cells [60,133]. In addition, as mentioned in the introduction, some specially structured NPs can act as the PSs by themselves, *e.g.*, Au NPs, silicon NPs, black phosphorus NPs, Carbon NPs or semiconductor polymers-based NPs [24,42]. Compared with traditional small molecule organic-based PSs, they possess gifted photoactive properties, such as the high ROS production ability and adjustable excitation light wavelength [62,134,135].

In this section, we first introduce how NPs enhance the current PSs treatment efficacy, and then discuss the relationship between the NPs and TMEs to show how to manipulate TME to provide a better therapeutic environment. Finally, we discuss how to circumvent the limitation of light penetration and improve light excitation efficiency by smart NPs design. Some of the latest related papers are chosen as typical examples.

#### 2.2.1 NPs as PDT agent carriers

Many traditional and typical organic PSs have been approved for clinical trials and applications [136,137]. However, as described above, to avoid unfavorable systemic distribution *in vivo* and improve PSs accumulation in the tumor, NPs are used as the nanocarriers to load and targeted deliver these PSs and other auxiliary agents to the tumor sites.

**Fig. 3:**
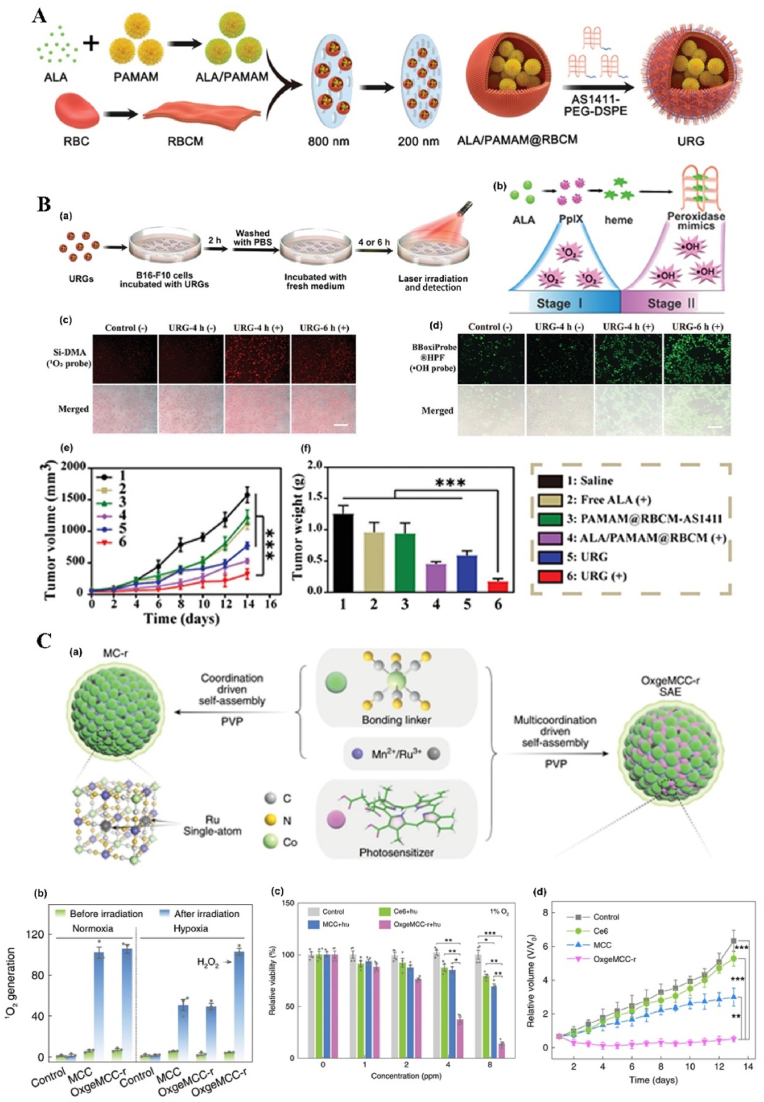
**NP-based innovative cancer PDT nanosystems. A. Schematic illustration of fabrication and antitumor effect of the URG. B. Uninterrupted ROS generated in B16–F10 cells and *in vivo* evaluation of antitumor efficacy**: (a) Schematic representation of treatment procedure for B16–F10 cells; (b) Illustration of uninterrupted ROS generation in B16–F10 cells; (c) Fluorescence imaging of ^1^O_2_ in B16–F10 cells at different times after URG treatment; (d) Fluorescence imaging of H_2_O_2_ in B16–F10 cells after URG treatment; (e) B16–F10 tumor growth curves of all treated groups (n = 6); (f) weights of the collected B16–F10 tumors at day 14 (n = 6). Adapted from Ref. [138]. Copyright *©* 2022, Wiley-VCH. **C. Photodynamic profile of OxgeMCC-r NPs**: (a) schematic illustration of OxgeMCC-r; (b) ^1^O_2_ generation under different conditions before and after 671 nm laser irradiation (100 mW/cm^2^, 30 s); (c) cell viability assay of free Ce6, MCC, and OxgeMCC-r SAE treated 4T1 cells in hypoxic conditions under 671 nm light irradiation; (d) Relative 4T1 tumor volumes of mice after various treatments (control, Ce6, MCC, and OxgeMCC-r, *n* = 5). Adapted from Ref. [150]. Copyright *©* 2020, Springer Nature.

NPs can deliver PSs and cooperate with PSs in ROS production. For example, protoporphyrin IX (PpIX) can easily and rapidly switch into non-photoactive heme when they bind with Fe^2+^ ions in the mitochondria, which results in the undesirable pause on the production of the ROS, followed by the therapy interruption. To solve this problem, Shi et al. fabricated an “uninterrupted ROS generator” (URG) NPs which consist of the 5-aminolevulinic acid-polyamidoamine (ALA-PAMAMs), red blood cell membrane (RBCM) and DNA aptamer-AS1411 with G-quadruplex (Fig. 3A and B). In the NPs, the biocompatible and flexible RBCM behaved as the nanocarrier of the PDT agents and increased the NPs blood circulation and stability; ALA-PAMAMs worked as the precursor of PpIX and increased the accumulation of PpIX in mitochondria; and AS1411 functioned as the target to the tumor cells and the ligand for the intracellular self-assembly with heme. URG NPs can transfer the generated heme into a functional enzyme catalyzing the H_2_O_2_, overproduced in the tumor cells, into hydroxyl radicals (·OH) after the PS's conversion and even post light irradiation, ensuring the continuity of the PDT therapy [138]. Another typical example is the semiconducting polymer-based PSs nanocarrier. Besides the super photostability and high biocompatibility, the semiconducting polymer NPs can be easily surface functionalized or facilely conjugated with biomolecules for different purposes, *e.g.*, for larger absorption range or loading capacity [55,118,139]. Tang et al. developed a nanocarrier based on the photoreactive oxetane groups modified semiconducting polymer to dope with Chlorin e6 (Ce6), which was called Ce6-doped semiconducting polymer dots (Ce6-Pdots). After UV photo-crosslinking, a polymeric network was formed due to the reactions between the modified side chains to prevent the retained Ce6 leakage. Because the emission range of the nanocarrier overlaps with the Ce6 absorption range, upon green light (520 nm) irradiation, the semiconducting polymer can transfer the excitation energy to the Ce6 and then efficient ^1^O_2_ were generated. Compared with Ce6 alone, the energy transfer process can avoid the relatively high dose of light irradiation and due to the large absorption cross section of the semiconducting polymer-based nanocarrier, the ^1^O_2_ generation was amplified, which resulted in highly effective *in vitro* PDT effect even with low NPs concentration (10 μg/mL) and light dose (60 J/cm^2^) and the *in vivo* tumor growth was apparently inhibited after the PDT treatment [140]. NPs can also protect PSs from inactivation and degradation in the complex biological environment. For example, ICG, which is a PSs approved by U.S. Food and Drug Administration (FDA) for clinical treatment and imaging, still suffers from the instability in aqueous solution, which hinder its PDT efficacy. To improve its therapeutic effect, Yang et al. fabricated ICG-oxygen nanobubbles (named as ICG-NBs-O_2_) through assembling the free ICG molecules with NBs-O_2_. The NPs assembly occurred on the gas-liquid interface due to the hydrophilic-hydrophobic interaction. Compared with free ICG, the ICG-NBs-O_2_ NPs demonstrate better aqueous stability, which kept 64% of the initial loaded ICG after 4 days. Meanwhile, the quantum yield (QY) of the generated ROS, increased up to eight times compared with free ICG solution. With those enhanced stability, QY and loaded oxygen contents, ICG-NBs-O_2_ NPs showed outstanding biosafety and PDT therapeutic effect, both *in vitro* and *in vivo* [141]. In addition, if the nanocarriers themselves based on the self-degradable skeleton matrix, the degradation of the NPs can be further help resolve the ROS depletion in the carriers and avoid the possible toxicity of the excessive PSs [59,142,143]. For example, Hung et al. fabricated a conjugated polymer skeleton-based NPs for the tumor PDT. The polymers consist of the PSs-AIE monomer (TPA-yne)-and the conjugated imidazole units through the Sonogashira coupling reaction and then the conjugated polymers were encapsulated into the pluronic F127 as the NPs core with the nanoprecipitation way. During the white light irradiation, the ROS (superoxide radical) can be produced for the PDT treatment and also caused the self-degradation of the polymers, which helps to avoid the possible phototoxicity of the residual PSs after the PDT [143].

#### 2.2.2 Hypoxic TME-responsive NPs

As a result of the abnormally rapid growth of the tumor tissues and the resulting vascular growth malformations, a hypoxic environment is found within the tumor. However, for ROS generation during PDT, both Type I and Type II mechanisms rely on to oxygen to different degrees, which means the initial hypoxia and the oxygen consumption during the treatment both limit the effects of the PDT. To address these limitations, novel hypoxic TME-responsive NPs are increasingly introduced into the cancer PDT to modulate TME. The typical modulations include increasing TME oxygen concentration, reducing the tumor cells oxygen consumption, consuming the excessive glutathione (GSH) or normalizing the tumor blood vessels.

To solve the oxygen-deficiency in the TME, the most direct solution is to deliver oxygen directly into the tumor tissue as introduced in Section 2.2.1. For example, Liang et al. reported a nanocarrier through ultrasonically dispersing the perfluorooctyl bromide (PFOB) liquid with excellent oxygen solubility into the porphyrin grafted lipids (PGL) NPs with ∼39% porphyrin loading efficiency and the followed oxygen encapsulation. The fabricated O_2_@PFOB@PGL NPs showed outstanding oxygen loading and package stability because of the high PFOB encapsulation (up to ∼98%) brought by the powerful hydrophobic interactions between the PFOB and PGL. This structure facilitates the effective intratumorally co-delivery of the oxygen and PSs. The fluorescent self-supplemented O_2_@PFOB@PGL NPs showed excellent capacity of ROS generation under 650 nm laser irradiation and also showed the capacity as a computed tomography (CT) imaging contrast agent, which guaranteed the powerful PDT can be carried out under the dual modality imaging guidance. The experimental results indicated that the O_2_@PFOB@PGL NPs effectually alleviated the hypoxia, generated abundant ROS, and subsequently downregulated the COX-2 expression. The *in vivo* experiment showed that the HT-29 colon tumor and the liver metastasis were dramatically inhibited by the O_2_@PFOB@PGL NPs-based PDT [144].

In the hypoxic TME, the overproduced hydrogen peroxide (H_2_O_2_) caused by the abnormally consistent oxidative stress is another common feature. It has been proved that H_2_O_2_ could be used as an effective endogenous source of oxygen to alleviate hypoxia, when it is catalyzed or decomposed by various NPs, especially by manganese oxide (Mn_x_O_y_)-based NPs [[145], [146], [147], [148]]. Recently, *Zhu* et al. developed a biocompatible and theranostic nanoformulation which consisted of the co-encapsulated Ce6, manganese dioxide (MnO_2_) and the engineered nanocarrier, ferritin (Ftn). The experimental results demonstrated that the fabricated Ce6/Ftn@MnO_2_ NPs with 15.5 nm uniform size can pronouncedly accumulate in the tumor because of the high affinity of the Ftn to the transferrin receptor 1, which is overexpressed on many cancer cells. The intratumoral NPs worked like a nanozyme, catalyzing the endogenous H_2_O_2_ decomposition to produce the additional O_2_ for the hypoxia relief, which significantly decreased the expression of hypoxia-inducible factor (HIF)-1α. Under 660 nm laser exposure, compared with free Ce6, the PDT anti-tumor efficacy of the NPs exerted much better tumor inhibition with negligible normal tissues damage, which can be ascribed to the improved retention of Ce6 and adjusted TME suitable for the PDT. Moreover, the fluorescence of the Ce6 and the manganese ions from the acidic degradation of MnO_2_ endows the Ce6/Ftn@MnO_2_ NPs with the fluorescence and Magnetic Resonance Imaging (MRI) capacity to be tracked during the whole treatment process [149].

In addition to the manganese oxide-based NPs, manganese ion-based NPs, especially the manganese ion-based MOFs NPs, have been developed as nanozymes for H_2_O_2_ decomposition and PDT, due to its brilliant ability as the *T1*-weighted MRI contrast agent, low toxicity, and immunity enhancement properties. Recently, Wang et al. fabricated self-assembled nanozyme, called OxgeMCC-r-single atom enzyme (SAE), through encapsulating Ce6 into the single-atom Ru-anchored Mn_3_[Co(CN)_6_]_2_. Compared with MnO_2_-based PDT nanozymes, the Ru of the OXgeMCC-r SAE can rapidly and durably catalyze the decomposition of the endogenous H_2_O_2_ into O_2_ without NPs self-consumption, while the MRI capability still maintained. Moreover, the porous structure form the manganese ion-based MOFs endowed high Ce6 loading efficiency, up to ∼30 wt-% loading capacity and ∼76% loading efficiency calculated from the Ce6 adding amount of 60 mg. The *in vivo* results demonstrated that the OXgeMCC-r SAE can effectively alleviate the hypoxic environment in the solid tumor with the catalyzed H_2_O_2_ degradation, enhancing the generation of ROS and resulting in apoptotic cancer cells death under the 671 nm laser irradiation. The theranostic OXgeMCC-r SAE fabrication could progress the development of the single-atom nanozymes in the PDT study (Fig. 3C) [150].

In addition to oxygen generation, NPs also haven been developed as “reducing expenditure” tool to relive the hypoxia by decreasing the cancer cells respiration (oxygen consumption). Specifically, NPs loaded with cellular metabolism regulation agents can interfere with the oxygen consumption and adenosine triphosphate (ATP) production, which alleviates the hypoxic TME and reduces the occurrence and mortality in some tumor cases. Recently, for efficient intratumoral delivery and controlled release of the 1-dimethyl biguanide hydrochloride (Metformin or Met) and PT agents, Meng et al. reported a gelatin-based multifunctional nanoreactor. This nanoreactor, MCGPD ∼ RGN NPs, was constructed by loading Met and Ce6 on the gelatin NPs. Originally used as a commonly clinical type-2 diabetes mellitus-hypoglycemic drug, Met has been found to stimulate the 5’ adenosine monophosphate-activated protein kinase (AMPK) and inhibit mitochondrial respiratory chain complex I. The nanoreactor was further coated by PDA, followed by DOX absorption and Arg-Cly-Asp (RGD) peptide modification, which endowed the NPs active targeting ability to the cancer cells and improved the intratumoral NPs accumulation. The experimental results indicated that the released Met, induced by the TME overexpressed matrix metalloproteinase-2 and PTT, can elevate the oxygen content for the 660 nm laser-trigged PDT and also can decrease the ATP production for the heat shock proteins (HSPs)-resisted PTT, effectively leading to the cells apoptosis and tumor growth inhibition both *in vitro* and *in vivo* [151].

Apart from Metformin, atovaquone (ATO), originally used for antimalaria and *anti*-pneumocystis pneumonia, has also been discovered to be a cellular oxygen consumption reducing agent through inhibiting mitochondrial complex III. Fan et al. established the dual-drug NPs by encapsulating two FDA approved drugs, verteporfin (VER) as PSs and ATO as mitochondria respiratory inhibitors, into the PLGA-block-PEG methyl ether. After the intravenous injection, the dual-drug NPs can reach to the tumor sites by enhanced permeability and retention (EPR) effect and the VER targeting ability, which led to the effective intratumoral co-delivery of the PSs and hypoxia alleviator. Experimental results demonstrated that based on the hypoxia relief and laser exposure, no matter *in vitro* or *in vivo*, the fabricated dual-drug NPs exhibited potent PDT effect against cancer [152].

An alternative method to enhance the PDT effect is to deplete the excessive TME GSH with NPs due to the redox balance disruption. Ruan et al. synthesized methylene blue-loaded Cu-tryptone (Cu-Try/MB) NPs with a green method. In the fabricated Cu-Try/MB NPs, the Cu-tryptone can consume the intracellular GSH through redox reaction between Cu and GSH, which can increase the existing ROS level in the tumor, and MB used as the PSs can generate ROS when irradiated with laser. The *in vitro* and *in vivo* results indicated that under 650 nm laser irradiation, the cancer cells can be killed effectively by the enhanced PDT accompanied with the GSH-reducing NPs [153].

Another recent publication combined H_2_O_2_ consumption with GSH depletion to modulate tumor TME for PDT. Zeng et al. fabricated dual-modal imaging guided and biodegradable NPs by encapsulating the hydrophobic pro-PSs (MBPB, MB incorporated with a p-phenylboronic ester (PB)), into bovine serum albumin (BSA). The experimental results indicated that after the uptake by the cancer cells, the BSA-MBPB NPs can be activated by the endogenous H_2_O_2_, releasing the MB as the PSs for the ROS generation, and the by-products of the activation, quinone methide, can react with the intracellular GSH to boost the ROS produce in the synergistic manner, enhancing the PDT effect under the laser irradiation. Moreover, during the intracellular transformation from MBPB to MB, the fluorescence of the MB recovered and the based on the absorption responsiveness between the BSA-MBPB and H_2_O_2_, PAI was also induced for the tumor region detection [[154], [155], [156], [157]]. The fluorescent/PA dual-modal imaging signal provided precise location guidance for the following laser irradiation. The fabricated BSA-MBPB NPs provided a highly efficient and accurate PDT for the cancer treatment [158].

#### 2.2.3 Light excitation-enhanced NPs

Since the irradiation of the light on the PSs is the trigger of the whole PDT, it is critical to ensure sufficient light excitation of PSs to achieve satisfying PDT outcomes. There are several independent factors affecting the light excitation efficiency, such as the light exposure dose, light fluence rate, light penetration depth and interval time between the PSs injection and irradiation [23]. The penetration depth is dependent on the light wavelength, which has to match the absorbance window of the PSs. But for most of the PSs, typically exampled by porphyrin-family ones, their main light absorption locates in the relative shorter wavelength (UV–Vis range). Within this range, the penetration depths are less than 2 mm [159,160] and it may lead to insufficient irradiation of the PSs. To address this problem, two strategies are currently under investigation. One is to use the PSs with NIR-absorbance window (650–1350 nm) and the another is introducing the NPs with capacity to “deliver deep light” into PDT system, such as upconversion NPs (UCNPs) [[161], [162], [163]], two-photon excitation NPs [60,164] and persistent-luminescence-based NPs (PLNPs) [[165], [166], [167]].

To overcome the limited light penetration depth of the Ce6 excitation light and enhance the oxygen content in the TME for better PDT efficacy, Liang et al. fabricated UCNPs-based multifunctional nanocarrier, UCNPs@G4/Ce6/CAT-CTPP, to co-deliver the PSs and H_2_O_2_ catalyst into the tumor site. The UCNPs@G4/Ce6/CAT-CTPP NPs were constructed by the 20%Yb, 2%Er@NaGdF4 (NaYF_4_) as the UCNPs core which converted the NIR light to visible light for Ce6 excitation. The core was coated by the fourth-generation hemispherical polyamide dendrimer (G4), which was covalently linked to UCNPs by thiol-ene and azide-acetylene click reactions, to load the Ce6 and catalase (CAT) and the 3-carboxypropyl triphenyl-phosphonium bromide (CTPP) as the mitochondria targeting molecules. The experimental results indicated that when the intratumorally accumulated UCNPs@G4/Ce6/CAT-CTPP NPs were exposed by the 980 nm laser, the UCNPs could convert the incident light into Vis red light (around 650 nm, within the maximum absorption of Ce6), and significantly stimulated the ROS generation synergized by the produced O_2_ from the H_2_O_2_ catalyzed by CAT. The combined therapeutic outcomes from Ce6 led to the most prominent tumor inhibition effect *in vivo* [168].

As a result of the excellent tissue penetration, large photon absorption cross-section and the capacity to emit the high-energy light, the two-photon excitation NPs-based PSs have been emerged as promising PDT agent for the cancer therapy [169,170]. Guo et al. fabricated the semiconducting polymer-polythiophene quaternary ammonium-based NPs, which was called PNPs, as the theranostic PSs for two-photon excited PDT. These NPs were prepared through facile one-pot synthesis, ultrasonicating the polythiophene quaternary ammonium and the 2-distearoyl-sn-glycero-3-phosphoethanolamine-N-[methoxy(polyethyleneglycol)-2000] and followed by the solvent evaporation. The characterization results indicate that the NPs can generate efficient ^1^O_2_ under two-photon excitation due to the high and similar ROS quantum yields no matter under 532 nm laser or 800 nm fs pulse laser. Moreover, the two-photon fluorescence images showed that the detection depth can be up to 2100 μm in mock tissue, which significantly increase the light excitation depth for the fluorescence imaging and PDT. The *in vitro* result showed that after the 6 h incubation and 10 min laser (800 nm) irradiation at the concentration of 500 μg/mL NPs, almost 80% cells died and *in vivo* results showed an evident tumor growth inhibition and prolonged mice survival after the two-photon excited PDT [171]. Compared with UCNPs-based NPs, PLNPs can store the excitation energy and then emit intriguing long-lasting luminescence, so they are not limited by the water absorption on the laser exposure. Furthermore, PLNPs can avoid the possible overheating and tissue damage from the longtime laser irradiation. Recently, Chang et al. fabricated a laser-free PDT nanosystem based on the two-dimensional PL materials, CaAl_2_O_4_:Eu,Nd nanosheets (CAOPLNSs) with blue persistent emission from the 5d-4f electron transition of Eu^2+^ and the existed electron traps upon UV irradiation, which worked as excitation light source. In addition to CAOPLNSs, the PDT nanosystem (CVT) contained the linked VER used as the PSs and surface-modified triphenylphosphine (TPP) used as mitochondrial-targeting molecules. The experimental results showed the large overlapping between the CAOPLNSs emission spectrum and the VER absorption spectrum, indicating the efficient capacity of the CAOPLNSs to excite the VER for the ROS generation. Moreover, the fabricated CVT featured with increased afterglow time and distinct PL, enabling the ROS continuous production during the PDT. Meanwhile, assisted by the disruption ability of the Nd^3+^ ions to the lysosome phosphoprotein membrane and the excessive mitophagosomes and autophagosomes production activated by the mitochondrial-targeted CVT, the PDT efficacy was amplified. Both *in vitro* and *in vivo* results demonstrated the extraordinary therapeutic performance of the CVT NPs, which could provide a new path for the PL-based PDT NPs [172].

We summarize recent representative NPs-based PDT work in Table 3, presented in terms of their functions in PDT, PSs, *in vitro* and *in vivo* therapeutic effects. In addition to the up-to-date papers we list above, readers are welcome to read earlier but more detailed and specific reviews about the NPs-based PSs or NPs-based PDT, the review from Lan et al. [42] and the review from Xie et al. [62] are recommended.

**Table 3:** NP-based PDT studies.^a^

Main function of the NPs	NPs	PSs	Light source	*In vitro* anti-cancer effect	Particles injection dosage of *in vivo* anti-cancer study	*In vivo* anti-cancer effect	Reference
Delivery carriers	Quinolinium conjugate (PQC)-based fiber-forming nanoPSs (PQC NF)	Pheophorbride A	*In vitro*: 633-nm LED array *In vivo*: 680 nm laser	OSC-3 cells IC_50_: 0.12 μM	1 mM (10 nmol per 50 mm^3^ tumor size)	OSC-3 tumor: The laser-treated PQC NFs exhibited the best antitumor efficiency, which achieved a 100% complete cure rate.	[173]
Delivery carriers	PFH@PEG-F_54_-BODIPY	Boron dipyrromethene amphiphile (BODIPY)	*In vitro* and *in vivo*: 660 nm	A375 cells >50% tumor cells were killed	BODIPY dose: 2 μmol/kg	A375 melanoma tumor: exhibited much slower tumor growth and 70% of mice survived 40 days	[174]
Delivery carriers	O_2_@PFOB@PGL	Porphyrin	*In vitro* and *in vivo*: 650 nm	HT-29 cells IC_50_: 0.011 ± 0.003 μM	200 μL (2 mg/mL)	HT-29 tumor: complete tumor elimination at the 26th day post treatment	[144]
Delivery carriers	SWCNTs-HA-Ce6	Ce6	*In vitro:* 660 nm	Caco-2 cells cell death ∼85% at 10 J/cm^2^ cell death 77% at 5 J/cm^2^	*N/A*	*N/A*	[175]
Delivery carriers	Amphipathic chimeric peptide-based spherical micelles	PpIX	*In vitro* and *in vivo*: 630 nm	4T1 cells COS7 cells Over half of 4T1 cells were found at the stage of early apoptosis or late apoptosis after irradiation for 30 s. An obvious phototoxicity against COS7 cells in an irradiation time-dependent manner	200 μL (1.2 mg/mL)	4T1 tumor: the tumor of the mice was obviously suppressed	[176]
Delivery carriers	@E7-ICG-BSA nanovaccines	ICG	*In vitro* and *in vivo*: 808 nm	bone marrow-derived dendritic cells (DCs) induced-maturation	100 μL (1 mg/mL)	Tc-1 cervical tumor significant inhibition of tumorigenesis, with smaller tumor sizes and tumor growth was effectively delayed.	[177]
Delivery carriers	ICG-NBs-O_2_	ICG	*In vitro* and *in vivo*: 808 nm	Cal27 cells significant cell killing ability	80 μL (equivalent ICG concentration: 0.1 mg/mL)	Cal27 tumor: The relative tumor volume gradually decreased to 0.56 of the initial tumor size	[141]
Delivery carriers	URG	PpIX	*In vitro* and *in vivo*: 532 nm	B16–F10 cells apoptosis rate: ∼55% and cell viability:∼18%	equivalent ALA at 20 mg/kg	B16–F10 tumor: the strongest inhibition of tumor growth, nearly 80% regression of tumors, and the most severe DNA damage, most severe damage to the tumor cells and the strongest apoptotic nuclear signals	[138]
Delivery carriers	Ce6-Pdots	Ce6	*In vitro* and *in vivo*: 520 nm	SCG-7901 cells almost all the cells were killed even with low concentration: 10 μg/mL and low light dose: 60 J/cm^2^	Intravenous injection: 100 μL (100 μg/mL) Intratumoral injection low dose: 100 μL (50 μg/mL) Intratumoral injection high dose: 100 μL (100 μg/mL)	SCG-7901 tumor: the tumor growth in all the PDT treatment groups were obviously lower than the control group and the tumor growth rate in intratumoral injection high-dose group was the lowest.	
Delivery carriers	Self-degradable conjugated polymer/F127 NPs	TPA-yne	*In vitro* and *in vivo*: white light	Hela and 4T1 cells the Hela cell viability: ∼20% without pre-irradiation the 4T1 cell viability: lower than 20% without pre-irradiation	200 μg/mL (25 μL per 50 mm^3^ tumor)	4T1 tumor The tumor volume in PDT group was almost steady and even diminished at the end and the H&E staining showed the tissue recovery only in PDT group	
TME-responsive NPs (H_2_O_2_-responsive)	Mn_3_[Co(CN)_6_]_2_	Ce6	*In vitro* and *in vivo*: 671 nm	4T1 cells Nearly 90% cancer cells were killed under hypoxic condition	100 μL (at a Ce6 concentration: 4 mg/kg)	4T1 tumor remarkable tumor suppression and average weight of tumor tissues was the lowest, at only 0.19 g	[150]
TME-responsive NPs (H_2_O_2_-responsive)	Ce6/Ftn@MnO_2_	Ce6	*In vitro* and *in vivo*: 660 nm	4T1 cells Cell proliferation was reduced to 3% at the high concentration	200 μL (20 mg/mL)	4T1 tumor: an evident tumor inhibition	[149]
TME-responsive NPs (H_2_O_2_-responsive)	BSA-MBPB	MB	*In vitro* and *in vivo*: 633 nm	HepG2 cells Cell viability was inhibited down to 37%	100 μL (50 μg/mL)	HepG2 tumor: the tumor volume shrunk persistently, and the tumor growth was almost completely inhibited after treatment for 18 days	[158]
TME-responsive NPs (H_2_O_2_-responsive)	PS-Pd@Pt nanosystem (Pd@Pt-PEG-Ce6)	Ce6	*In vitro* and *in vivo*: 808 nm (PTT) and 660 nm (PDT)	4T1 cells The phototoxicity of Pd@Pt-PEG-Ce6 was higher since Ce6 loaded on Pd@Pt-PEG could be ingested more by cells by 660 nm laser only and significant cell death by 808 nm laser and 660 nm laser	200 μL (1 mg/mL)	4T1 tumor: noticeable tumor growth inhibition in 12 d by 660 nm laser only and 808 and 660 nm laser irradiation group resulted in the most effective tumor growth inhibition and the tumors could be completely eliminated at the 6th day post injection	[178]
TME-responsive NPs (H_2_O_2_-responsive)	Hollow MnO_2_/DOX/BPQDs	black phosphorus QDs (BPQDs)	*In vitro* and *in vivo*: 808 nm (PTT) and 630 nm (PDT)	HepG2 cells the cell viability: ∼54% by 630 nm laser and the cell viability: ∼29% by 630 nm laser and 808 nm laser	200 μL (MnO_2_:10 mg/kg; DOX: 4.5 mg/kg; BPQDs:10 mg/kg)	HepG2 tumor: more obvious inhibitory effect of tumor, the smallest tumor size and weight, and tumor slices exhibited the maximum necrosis by 630 nm laser and 808 nm laser	[179]
TME-responsive NPs (H_2_O_2_-responsive)	Hollow-MnO_2_-PEG/Ce6&DOX	Ce6	*In vitro* and *in vivo*: 660 nm	4T1 cells the most effective in killing cancer cells by PDT-based synergistic therapy	200 μL (MnO_2_:10 mg/kg; SiO_2_: 25 mg/kg; Ce6:4.7 mg/kg; DOX: 4.5 mg/kg)	4T1 tumor: significant tumor growth-inhibition effect, the slowest growth speed and smallest volumes	[180]
TME-responsive NPs (H_2_O_2_-responsive)	IrP-losartan@V_2_O_5_	IrPVP	*In vitro* and *in vivo*: 635 nm	H22 cells IC_50_: 17.53 μg/mL under normoxic and IC_50_: 18.19 μg/mL under hypoxic conditions	Three times each time: IrPVP: 60 mg/kg; V_2_O_5_: 5 mg/kg; losartan: 8 mg/kg	H22 tumor significant tumor inhibition by PDT the best tumor inhibition effect by fractionated PDT	[181]
TME-responsive NPs (reduce oxygen consumption)	Zr- MOF@PPa/AF@PEG	Pyropheophorbide-a (PPa)	*In vitro* and *in vivo*: 670 ± 10 nm	HepG-2 cells Inhibition rate reached 98%	100 μL (equivalent PPa concentration: 0.8 mg/mL)	4T1 tumor: implanted tumors were atrophied and scabby	[182]
TME-responsive NPs (reduce oxygen consumption)	ATO and ICG-BSA loaded Gel NPs (Ato-ICGGNPs)	ICG	*In vitro* and *in vivo*: 808 nm	Hela cells Specifically, populations of cells undergoing late stage-apoptosis increased by 143.7-fold for Ato-ICG-GNPs	100 μL (ATO: 330.15 μg/mL; ICG: 37.44 μg/mL)	Hela tumor Persistent regression of tumor and the tumor was eliminated entirely after four times of PDT treatments	[183]
TME-responsive NPs (reduce oxygen consumption)	ATO/VER/PLGA-PEG	VER	*In vitro:*635 nm *In vivo:*685 nm	4T1 cells High lethality under hypoxic conditions	200 μL (VER: 1 mg/mL; ATO: 0.57 mg/mL)	4T1 tumors: complete elimination after treatment	[152]
TME-responsive NPs (reduce oxygen consumption)	MCGPD ∼ RGN	Ce6	*In vitro* and *in vivo*: 808 nm (PTT) and 660 nm (PDT)	MCF-7 cells cell viability: ∼26% under normoxia conditions, while the cell viabilities: ∼38% under hypoxia conditions	2 mg/kg (equivalent Ce6 content)	Breast tumor satisfactory antitumor effect when combining chemo-/PDT/PTT by 808 nm laser and 660 nm laser	[151]
TME-responsive NPs (reduce oxygen consumption)	TA-MSN@(*α*-TOS/ICG)-TPP	ICG	*In vitro* and *in vivo*: 808 nm	MCF-7 PDT group induced highly ∼84% of cell death and under hypoxic condition, still the highest lethality by 808 nm laser	2 mg/kg (α-TOS: 200 μg/kg; ICG: 100 μg/kg)	MCF-7 tumor: the tumor was gradually shrunken and even eliminated	[184]
TME-responsive NPs (reduce oxygen consumption)	DOX/Met/BSA-HA-Carbon dots(CDs)	CDs	*In vitro* and *in vivo*: 532 nm	MCF-7 cells and MCF-7/ADR cells the most effective therapeutic efficacy by PDT-based synergistic therapy	DOX: 5 mg/kg; Met: 15 mg/kg	S180 tumor: the best effective tumor growth inhibition efficacy by PDT-based synergistic therapy	[185]
TME-responsive NPs (reduce oxygen consumption)	PM-W_18_O_49_-Met	W_18_O_49_	*In vitro* and *in vivo*: 808 nm	Raji cells the lowest detected viability and the highest apoptosis rate	W_18_O_49_: 50 mg/kg; Met: 16 mg/kg	Raji lymphoma: dramatically decrease of the tumor volume and the largest necrosis and the fewest nuclei in tumor tissues	[186]
TME-responsive NPs (GSH-responsive)	PEG-terminated ZnTPPC6-based poly disulfide ester (PEG-b-PTPPDS-b-PEG)	Porphyrin	Light emitting diodes (LEDs) lamp	A549 cells IC_50_: 2.11 μg/mL	*N/A*	*N/A*	[187]
TME-responsive NPs (GSH-responsive)	Cu-Try/MB	MB	*In vitro* and *in vivo*: 650 nm	Hela cells the cell death rate reached 71%	200 μL (80 μg/mL)	U14 tumor: effectively control tumor growth	[153]
TME-responsive NPs (GSH/H_2_O_2_-dual responsive)	COF–Au–MnO_2_-HA	COF–Au–MnO_2_	*In vitro* and *in vivo*: 650 nm	4T1 cells mortality rate of was almost 80%	100 μL (1 mg/mL)	4T1 tumor: best antitumor efficacy	[188]
Light excitation-enhanced (UCNPs)	UCNPs@G4/Ce6/CAT-CTPP	Ce6	*In vitro* and *in vivo*: 980 nm	4T1 cells cell viability significantly lower	200 μL	4T1 tumor: obviously slower tumor growth and the most prominent tumor inhibition effect	[168]
Light excitation-enhanced (UCNPs)	DHyCUB	daunorubicin(DNR)	*In vitro:*980 nm	SKOV-3 cells cell viability: 25% MeWo cells cell viability: 58%	*N/A*	*N/A*	[189]
Light excitation-enhanced (UCNPs)	UR-Cyan	rose Bengal (RB)	*In vitro* and *in vivo*: 980 nm	4T1 cells Major population tumor cells were killed.	RB: 566 μg/mL; cyanobacteria: 7.2 × 10^8^ cell/mL	4T1 tumor: almost completely eradicated tumor xenografts in 5 days, overall tumor inhibition rate: ∼113%, the instant relative tumor inhibition: ∼198% on day 3	[190]
Light excitation-enhanced persistent-luminescence (PL)	CaAl2O4:Eu,Nd-PEG (CAP)+cyanobacteria-VER (Cb-VER)	VER	*In vitro* and *in vivo*: UV pre-excitation and white LED light re-irradiation	4T1 cells 60% cells death, early apoptosis:∼27% and later apoptosis: ∼39%	50 μL (CAP: 5 mg/mL)+50 μL (Cb: 5 × 10^7^ cell/mL; Vp: 0.5 mg/mL)	4T1 tumor: the distinct inhibition rate: ∼93%	[191]
Light excitation-enhanced (PL)	CAOPLNSs	VER	*In vitro* and *in vivo*: UV pre-excitation and white LED light re-irradiation	4T1 cells the lethality: 91%, cell viability: ∼11%	50 μL (5 mg/mL)	4T1 tumor: Significant suppression of tumor growth and high antineoplastic effects, the distinct inhibition rate: 96%	[172]
Light excitation-enhanced(two photon)	PNPs	PT2	*In vitro* and *in vivo*: 800 nm two-photon femtosecond pluse laser	Hela cells cell mortality rate: ∼80%	100 μL (500 μg/mL)	Hela tumor: evident tumor growth inhibition and no apparent tumor growth	[171]

a Abbreviations: 5-(4-(6-hydroxyhexyl) phenyl)-10,15,20-triphenylporphyrin (TPPC6-OH), 54 fluorine-19 (F54), perfluorohexane (PFH or PFC), porphyrin grafted lipids (PGL), single walled carbon nanotubes (SWCNTs), Human papillomavirus oncogenic protein (E7), trimethylammonium (TA), α-tocopherol succinate (TOS), mesoporous silica NPs (MSN), hollow mesoporous silica NPs (HMSNs), bis[2,4,5-trichloro-6-(pentyloxycarbonyl)phenyl] oxalate (CPPO), glucose oxidase (GOx), iridium(III) complex conjugated with hydrophilic poly(N-vinylpyrrolidone) (IrPVP), platelet membranes (PM), hybrid cubosomes loaded with up-converting NPs and daunorubicin (DHyCUB), human melanoma granular fibroblasts (MeWo), RB-encapsulated UCNPs onto cyanobacterial (UR-Cyan), polythiophene quaternary ammonium salt (PT2).

### 2.3 NPs-based photo-responsive drug release system

NPs-based internal or external stimuli-responsive drug delivery systems (DDSs) are considered as potential and efficient drug carriers to obtain triggered drug release in a controllable manner to avoid individual variability and drug leakage prior to reaching the target site [35,192,193]. For cancer treatment, compared with internal drug release-stimulus (e.g., GSH concentration and enzymatic activity of the tumor tissue compared to the healthy cells), external stimulus (e.g., light, magnetic field, and ultrasound) have been reported to have a better control over drug release [[194], [195], [196], [197], [198]]. In this section, we discuss photo-responsive drug release from nanomaterials and the main mechanisms behind them.

#### 2.3.1 NPs-based photothermal-responsive drug release

Compared with UV light or white light, the NIR is suggested for controlled drug release in recent years due to its safety and enhanced tissue penetration [193,199]. Upon exposure to NIR light, the drug release occurs due to the increased temperature, which can also cause cytotoxic effect on cancer cells in synergy with chemotherapy [[200], [201], [202]]. Photothermally active nanocarriers should have strong absorption in NIR range, temperature-responsiveness and demonstrate efficient tumor homing capacity in order to present desirable anticancer effect [203].

**Fig. 4:**
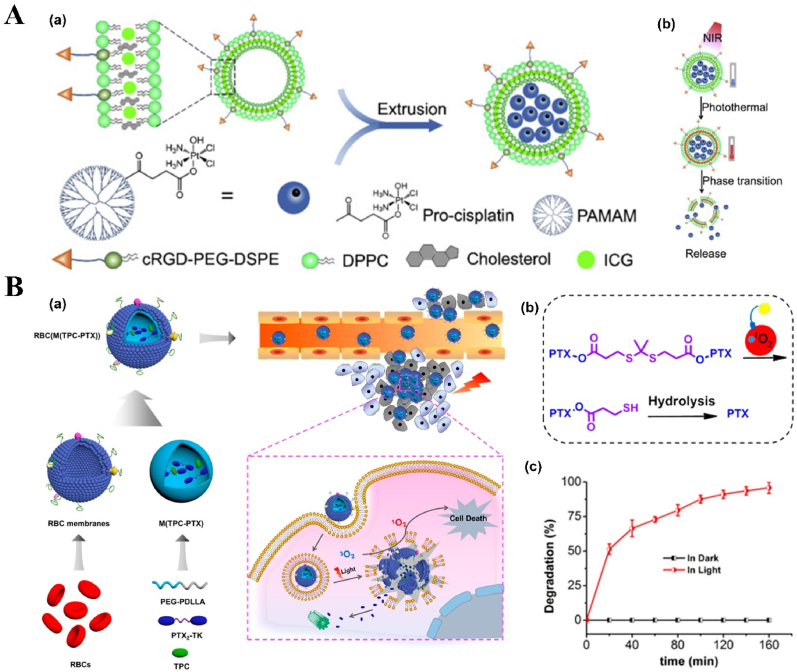
**NP-based innovative photo-responsive drug release nanosystems. A.** (a) Schematic illustration of PAM/Pt@IcLipo preparation; (b) Photo-responsive release of PAM/Pt NPs (<10 nm) under 808 nm laser. Adapted from Ref. [209]. Copyright *©* 2020, Elsevier. **B.** (a) Schematic illustration of synthesis and mechanism of RBCM-coated dimeric prodrug DDSs with NIR light triggered on-demand drug release; (b) Mechanism of ^1^O_2_ induced-PTX release and activation; (c) Degradation of PTX2-dithioketal by 638 nm laser irradiation (100 mW/cm^2^). Adapted from Ref. [216]. Copyright *©* 2018, American Chemical Society.

Different materials and nanostructures have been reported for this aim [199]. Among them, liposomes are one of the widely studied nanomaterials for photothermal responsive drug release [[204], [205], [206]]. For example, Zhu et al. reported a liposome composed of natural fatty acids, DOX and a NIR dye (IR780) for NIR-triggered drug release. In this work, photothermal responsive phase change particles with a melting point of 39 °C were prepared through using lauric acid and stearic acid. Irradiation with the 808 nm NIR light could induce IR780-mediated heat generation and melting of the liposome for drug release. This system could efficiently induce 90% of death on human lung A549 cancer cells, which was meaningfully higher than that without laser irradiation and demonstrated efficient photothermal-controlled chemotherapy [207]. Liposome is also used for the coating of porous nanomaterials to achieve photothermal drug release. In 2018, Li et al. reported that mesoporous carbon nanoparticle (MCN) were encapsulated into thermosensitive liposome bilayers for NIR based on-demand release of DOX from MCN and the liposome bilayer showed a phase transition temperature (Tm) of 40.7 °C. The drug could rapidly release upon 808 nm laser and the growth of 4T1 murine breast tumor in living mice was slower than that of mice without light exposure [208]. Another interesting capacity of liposome-mediated photo-responsive chemotherapy is designing novel nanosystems to improve the penetration of drugs into deeper areas of the cancer tissue for complete ablation of the tumor. For example, Xiong et al. reported a degradable liposome with potential to respond to NIR light, which was produced by the nanoprecipitation of cyclic arginine-glycine-aspartic acid (cRGD)-conjugated 1,2-Distearoyl-sn-glycero-3-phosphoethanolamine-*N*-[amino(poly(ethyleneglycol))] (DSPE-PEG), 1,2-dipalmitoylsn-glycero-3-phosphatidylcholine (DPPC), cholesterol, ICG, and cisplatin prodrug-grafted PAMAM dendrimer, called PAM/Pt (Fig. 4A (a)). ICG was incorporated in the bilayer of the liposome and PAM/Pt was loaded within the core of liposome to fabricate PAM/Pt@IcLipo. The final formulation showed a desirably long blood circulation time and an admirable targeting of the cancer tissue due to the surface cRGD ligand. ICG could increase the temperature of the particles under NIR laser irradiation at 808 nm to induce the destruction of PAM/Pt@IcLipo and release of ultrasmall PAM/Pt NPs (∼9 nm). Then the smaller PAM/Pt NPs could penetrate further into deeper area of the tumor tissue to exert effective chemotherapy (Fig. 4A (b)). This system resulted in the suppression of 4T1 tumor growth by 91% under 808 nm laser irradiation [209].

In addition to the photo-induced destruction of nanomaterials for drug release, phase change materials (PCMs) can be used in combination with hollow nanostructures to form photothermal responsive drug release formulations [210]. The photothermal hallow nanostratures can elevate local temperature under NIR light to induce the phase transition of PCM coating from a solid to a liquid and subsequent induction of drug release from the hallow particles. For example, Zhang et al. reported rod-based urchin-like Bi_2_S_3_ hollow NPs (named as U-BSHM) for the photothermal release of DOX. First, a sacrificial spherical template was used to synthesize U-BSHM with a photothermal conversion efficiency of ∼27%. U-BSHM was loaded with DOX and covered with 1-tetradecanol as a PCM agent that possesses a melting point of 38 °C. The drug release was enhanced by NIR irradiation at 808 nm and suppressed the viability of MDA-MB-231 cancer cells by synergetic action of PTT and DOX release. The temperature of the tumor tissue reached to nearly 49 °C under NIR laser irradiation *in vivo*, which was enough to induce the release of DOX and tumor eradication [211]. A similar concept has also been used by using 1-pentadecanol as PCM for the Prussian blue coated hollow iron oxide magnetic NPs loaded with chemotherapeutic DOX to release the drug at temperatures above 42 °C [212].

#### 2.3.2 NPs-based photodynamic-responsive drug release

It is feasible to integrate ROS-cleavable or hypoxia-cleavable moieties into a nanomaterial and fabricate photodynamic responsive carriers for drug release since the generated ROS and the oxygen depletion within tumor tissue during the PDT [213], which would allow to selectively release drug molecules within the tumor tissue [214,215]. For example, Pei et al. reported a RBCM coated nanocarrier loaded with photo-cleavable linker and dimeric prodrug (Fig. 4B (a)). In this work, tetraphenylchlorin was embedded in the inner core of the RBC membrane and could generate ROS under a 638 nm laser irradiation, which cleaved paclitaxel (PTX) from the dithioketal linkers. PTX could finally convert to its original chemical structure through hydrolysis and separation of the thiol group from its structure (Fig. 4B (b-c)) [216].

^1^O_2_ sensitive bis-(alkylthio)alkene (BATA) linker has also been reported to construct photodynamic-responsive drug release for on-demand chemotherapy [217]. For example, Yang et al. fabricated a novel light-responsive drug delivery platform based on Ce6 and mesoporous silica nanorods (named as CMSNRs). In this work, Ce6 doped CMSNRs were loaded with DOX, or larger cargos such as cis-Pt(IV) prodrug conjugated third generation dendrimer (G3-Pt). BSA was also coated on it via the BATA linkers and then modified with PEG. It was shown that a 660 nm light irradiation at a low power density of 5–50 mW/cm^2^ could induce^1^O_2_ generation by Ce6 and subsequent cleavage of the BATA linkers. This resulted in the separation of BSA-PEG from the surface of nanocarriers and drug release [218].

ROS-responsive diselenide bond (Se–Se) are also used for photodynamic-based drug release [[219], [220], [221]]. For example, Han et al. reported an efficient method for tuning the self-assembly of diselenide-containing block copolymers using red light. In this work, the co-encapsulation of porphyrin and DOX via diselenide bond inside a micelle could result in ^1^O_2_ generation under light (600–780 nm) irradiation and the cleavage of diselenide bonds, resulting in the disruption of micelles and on-demand DOX release. Aminoacrylate is another chemical group that has attracted attention for photodynamic-based drug release due to its ROS sensitivity. A copolymer was synthesized using PEG, poly-ʟ-glutamic acid and β-cyclodextrin (β-CD). Adamantane-conjugated PTX (Ada-PTX) and adamine-conjugated aza-BODIPY (Ada-BODIPY) were used as the prodrug guest molecules and the photosensitizer, respectively. A supramolecular drug delivery system was formed by strong interaction between β-CD and the adamantane units. 660 nm light could generate ROS and induce drug release by the cleavage of ROS-sensitive aminoacrylate groups in Ada-PTX [222]. As a result of the severer hypoxia in TME caused by the consumption of PDT, hypoxia-sensitive moieties have been also used to fabricate responsive drug release nanomedicines. For example, Qian et al. reported light-activated hypoxia-responsive drug-delivery nanosystem. In this work, ROS generating and hypoxia-responsive 2-nitroimidazole-grafted polymer was used to prepare DOX loaded NPs (termed as DOX/CP-NI) by a double-emulsion-based solvent evaporation/extraction method. A laser irradiation at 635 nm could lead to the ^1^O_2_ generation by the consumption of the dissolved oxygen in the TME. This led to a severe hypoxic microenvironment, which resulted in converting hydrophobic 2-nitroimidazole groups in the CP-NI to hydrophilic 2-aminoimidazoles and drug release by the dissociation of the particles. The responsive drug release in this study led to the strongest inhibition of the HeLa tumors compared with other groups [223].

Besides the ROS-responsive chemodrug release, the ROS-responsive linker also can be used to release the drug to modulate the TME for better PDT effect. For example, because the semiconducting polymer NPs has excellent capacity to produce the ROS under NIR irradiation, Zeng et al. reported a semiconducting polymer-based nanoenzyme conjugated with kynureninase via ^1^O_2_ cleavable linker through the bioconjugation. After 808 nm irradiation, the generated ^1^O_2_ not only caused the PDT-induced tumor cells-death, but also released the conjugated kynureninase, resulting in the degradation of the immunosuppressive kynurenine. These treatment effects finally led to the strong antitumor immunity, achieving the photodynamic/immuno combined therapy. The *in vivo* results indicate that the synergistic therapy can inhibit both the primary and distant tumors with the best therapeutic results, and prolong the tumor-bearing mice survival [134].

Although these studies for photodynamic-responsive drug release are promising, they still suffer from certain drawbacks that need to be overcome. For example, these systems may result in off-target drug release because of responding to endogenous ROS or hypoxia in other biological tissues rather than the target site of the interest, which causes side effects.

This section summarizes the recent approaches for photo-mediated drug release from different particles by different mechanism. More combined PTT/PDT/chemotherapy studies and their anticancer results will be introduced in Section 3.

## 3 PT-based combinatory cancer therapy

In Section 2, we discussed the development of the enhanced PDT or PTT based on NPs, and recent published studies about innovative particles design, which demonstrate how NPs enhance cancer PT treatment efficiency. However, in order to achieve better clinical treatment effect, in current NPs-based cancer treatment research, a growing number of studies have gradually focused on the combination therapy effect and this trend is also reflected in the PT-related explorations [[224], [225], [226]]. Here we will list several recent distinctive studies to demonstrate the remarkable synergistic therapeutic effect of the NPs-PT-based combination treatment.

### 3.1 PTT combined with PDT

The combination of PTT and PDT has confirmed to produce synchronous and synergetic anti-tumor effect though different mechanisms, leading to tumor cell apoptosis, necrosis, and the activation of immune clearance system [227,228]. The synergetic therapeutic outcomes could be ascribed to photothermal alleviation of tumor hypoxia though improving blood flow, thus enhancing photodynamic effect [229]. In the combination therapy, NPs can endow multiple functions including live imaging [[230], [231], [232]], tumor targeting [29,233,234], and TME-/thermal-responsive drug release [98,235,236]. These functions make it possible to achieve theranostics and enhance therapeutic efficiency.

**Fig. 5:**
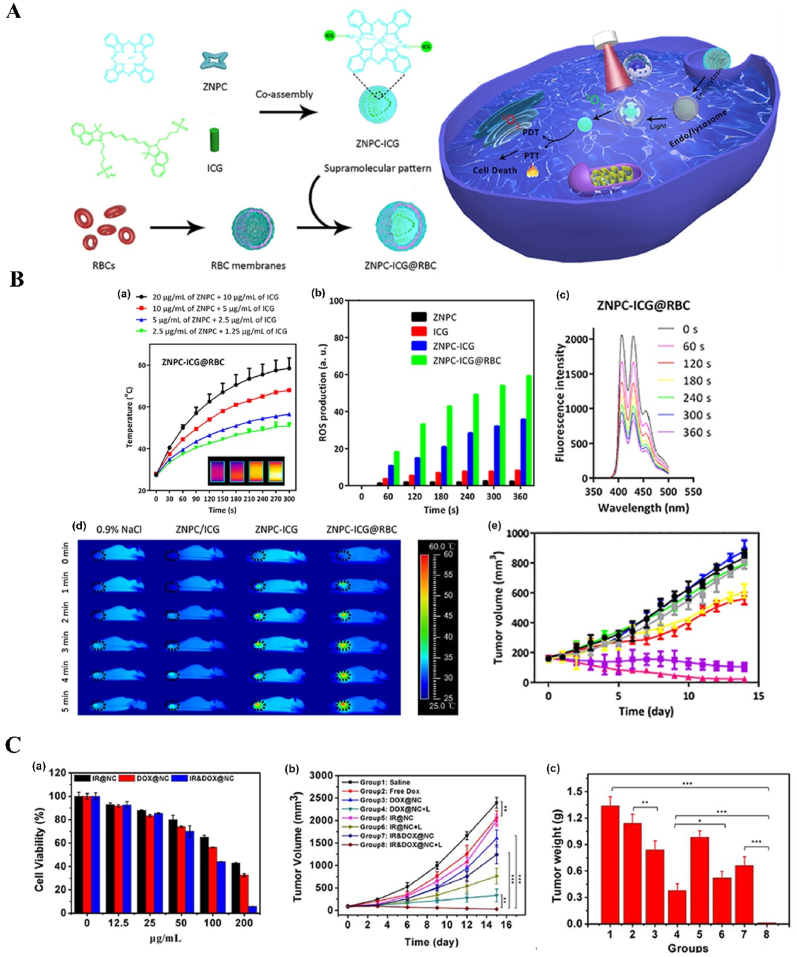
**NP-based innovative combined PTT/PDT nanosystems and NPs-based innovative combined PTT/PDT/chemotherapy nanosystems. A. Schematic illustrations of ZNPC-ICG@RBC formation and its enhanced PTT/PDT combination therapy for cancer treatment by using a single laser source. B. Photothermal and photodynamic properties of ZNPC-ICG@RBC NPs and *in vivo* combinatory anticancer effect**: (a) Temperature variation curves and infrared thermographic images of ZNPC-ICG@RBC NPs at different concentrations upon a 680 nm laser irradiation for 300 s; (b) ROS production rate; (c) Fluorescence intensity variation of ^1^O_2_ probe; (d) *In vivo* infrared thermal imaging of HeLa tumor-bearing mice after intravenous injection of the NPs; (e) Tumor volume changes of HeLa tumor-bearing nude mice treated with different NPs with or without 680 nm laser irradiation during 14 days. Adapted from Ref. [237]. Copyright *©* 2021, Elsevier. **C. *In vitro* and *in vivo* anticancer effect of the IR&DOX@NC NPs**: (a) Cell viabilities of 4T1 cells treated with different NPs under 808 nm laser exposure for 4 min at a density of 1 W/cm^2^; (b) Tumor volume growth curves of 4T1 tumor-bearing mice treated with different ways; (c) Tumor weight of 8 groups mice on day 15 after different treatments. Adapted from Ref. [251]. Copyright *©* 2021, Elsevier.

Chen et al. reported the erythrocyte membrane bioengineered NPs, ZnPC-ICG@RBC, with ICG and zinc phthalocyanine (ZnPc) as co-assembled core (Fig. 5A). The shape of co-assembly was adjusted from spindle-like to spherical shape depending on the ICG/ZnPc ratio. The erythrocyte membrane greatly prolonged the circulation time up to 72 h, and the tumor targeting ability was increased, doubling the NPs accumulation in the tumor tissue. Compared with the free ICG or free ZnPc, ZNPC-ICG@RBC nanoprobes exhibited superior photostability and excellent photothermal performance and photodynamic performance. In addition, with the RBC coating, the dispersity and physiological stability of the ZNPC-ICG were improved and the RBC membrane prevented the ZNPC-ICG contact and excessive aggregation, which all reduced the ROS quenching situation and enhanced the PDT efficiency. The *in vitro* results indicated that after the combination therapy, the cell viability in NPs group reduced to 10% and then the *in vivo* anti-tumor study showed that in ZNPC-ICG@RBC with laser irradiation group exhibited the best therapeutic effects, with complete tumor eradication (Fig. 5B) [237].

Liu et al. reported light-driven endogenous water oxidation-based nanomushroom, Ag–AgCl@Au NPs, for cancer combination therapy. Under visible light irradiation, the Au nanostructures were preferentially photo-deposited at the protuberant sites of the Ag–AgCl nanocubes and then on the surface of the AgCl nanocubes, forming the close Schottky contact. This structure and the plasmon effect significantly improved the utilization efficiency of photocarriers, which endowed the Ag–AgCl@Au NPs excellent photocatalysis ability to decompose the water into oxygen under NIR II light irradiation, alleviating the TME hypoxia. Meanwhile, the AgCl nanocubes also can drive the activated electrons in the conduction band to react with oxygen, producing the ROS for PDT. In addition, the photothermal conversion efficiency Ag–AgCl@Au NPs was as high as ∼73% due to the close contact between the Au and the semiconductor. The *in vitro* results showed that under 1064 nm laser irradiation, Ag–AgCl@Au NPs could overcome hypoxia and generate ROS for the PDT. With the synergistic effect of the simultaneously produced ROS and thermal effect, the IC_50_ value of combined PDT/PTT was 20 μg/mL, much lower than that of PDT alone (30 μg/mL) or PTT alone (80 μg/mL). Additionally, the combination index of the PDT and PTT was 0.92, further proving the combination effect toward killing the cancer cells. The *in vivo* results indicated that under 1064 nm irradiation, compared with other groups, Ag–AgCl@Au NPs group exhibited better inhibitory effect on the tumor growth and the H&E staining results showed the obvious cancer cells necrosis in the tumor section [238].

More representative NPs-based PTT combined with PDT studies are presented in Table 4.

**Table 4:** NP-based combined PTT/PDT.^a^

The NPs	PT agents	PSs	Laser (nm)	*In vitro* anti-cancer effect	Particles injection dosage of *in vivo* anti-cancer study	*In vivo* anti-cancer effect	Reference
Ce6-loaded MoS_2_-PEG	MoS_2_	Ce6	808/660	4T1 cell viability: less than 20%	Ce6: 2 mg/kg, MoS_2_-PEG: 6.85 mg/kg	4T1 tumor volume smaller than 140 mm^3^	[239]
AuNR/ICG/PEG-PCL	ICG and Au nanorods	ICG	785	About 15% of PC3 tumor cells underwent apoptosis under 3 min' light excitation	7.5 mg/kg (equivalent ICG content)	60% PC3 tumor-bearing mice were cured.	[240]
NaGdY_4_-UCNP@BSA-RB&IR825	IR825	RB	808/980	4T1 cell viability: less than 10%	20 μL (10 mg/mL)	4T1 tumor volume less than 50 mm^3^	[241]
Gd_2_O_3_@PPy/AlPc-HA	PPy	AlPc	808/670	4T1 cell viability: 20%	20 μL (2 mg/mL)	Combined PTT/PDT demonstrated the best therapeutic efficiency for 4T1 tumor, better than any single therapy	[242]
MnO_2_@Ce6@PDA-FA	PDA	Ce6	808/660	MCF-7 cell viability:10%	MnO_2_: 10 mg/kg, Ce6: 5 mg/kg	The most pronounced MCF-7 tumor growth inhibition in mice was observed upon combined therapy	[235]
PFOB @IR780& mTHPC@ NAcHis-TPGS modified liposome	IR780	mTHPC	808/660	TRAMP-C1 cell viability: less than 10%	mTHPC: 2.43 mg/kg, IR 780: 2.5 mg/kg	TRAMP-C1 tumors remained essentially the same size with the combination therapy, the full inhibition of tumor growth	[243]
MoO_3-x_-Ag-PEG-MnO_2_	Ag nanocubes	MoO_3-x_	808	HeLa cell viability less than 20%	20 μL (5 mg/mg equivalent [Mo] concentration)	HeLa tumor was completely ablated	[244]
17AAG@ P(2PMI-AQ)	2PMI-AQ	2PMI-AQ	660	4T1 cell viability: less than 20%	10 mg/kg	The most efficient suppression of 4T1 tumor growth	[245]
MSNR@Au-TPPS_4_(Gd)	Au	TPPS_4_(Gd)	808/660	4T1 cell viability: less than 30%	No dose information were provided.	The best treatment effect on 4T1 tumors under combination therapy compared with any single therapy	[246]
[PHC]PP@HA NPs	PDA	Ce6	808/670	PC-3 cell viability: less than 10% both in normoxia and hypoxia	4 mg/kg (equivalent Ce6 content)	The best PC-3 tumor inhibition, close to 100% of tumor inhibition rate and the tumor growth curves remained unchanged within 20 days	[247]
MC/MnO_2_/Ce6/CCM	MC	Ce6	808/660	4T1 cell viability: 25%	Four times injections: each time: 5 mg/kg (equivalent Ce6 content)	The best 4T1 tumor suppression rate: 93% and had the highest apoptosis or necrosis	[59]
MC/MnO2/Ce6/PEG/iRGD	MC	Ce6	808/660	4T1 cell IC_50_: 0.843 μg/mL	5 mg/kg (equivalent Ce6 content)	The best therapeutic effect on the 4T1 tumor and some 4T1 tumors were completely ablated	[58]

a The undefined abbreviations in the table: folic acid (FA), metatetra(hydroxyphenyl)chlorin (mTHPC), aluminum phthalocyanine (AlPc), allylamino-17-demethoxygeldanamycin (17AAG), two perylene monoimide moieties-diamino anthraquinone (2PMI-AQ), 5,10,15,20-Tetrakis (4-sulfonatophenyl)-porphyrin (TPPS_4_), PDA-hemoglobin-Ce6 (PHC), Mesoporous carbon (MC).

### 3.2 PTT, PDT combined with chemotherapy

The combination of phototherapy with chemotherapy has long been investigated. Compared with the PTT and PDT combination therapy, the chemotherapy drugs released from the PTT/PDT/chemotherapy nanoplatform could inhibit the regrowth of the damaged tumor blood vessels and eliminate the remaining cancer cells that survived after the cancer PTT or cancer PDT, especially after cancer cells develop thermal resistance. Chemotherapy drug also can prevent the possible tumor recurrence during the intervals between the cancer PT through the chemotherapy and elicit anti-tumor immune responses. Moreover, as we introduced in 2.3, NPs-based photo-induced drug release can improve the chemotherapy drug accumulation in the tumor and reduce the side effects of the chemotherapy, achieving the purpose of precise treatment. Furthermore, from a clinical point of view, because it is hardly possible to irradiate the whole body with appropriate irradiation doses, especially with the NIR laser, cancer PT faces huge obstacle to cure the advanced disseminated cancers (metastases). Thus, the combination therapy based on PT and chemotherapy exhibit great clinical promise on the clinical metastatic cancer treatment [16,201,248,249].

In order to enhance the multi-therapeutic effects, Feng et al. fabricated a nanoplatform with the capacity to produce the oxygen in TME, called Ini@PM-HP, which consists of a porous MOF (named as PCN-224(Mn)), a poly (ADP-ribose)polymerase (PARP) inhibitor(Iniparib), and the hyaluronic acid (HA) modified with PDA (HA-PDA). The HA on the surface endows the nanoplatform the capacity to bind to the HA receptor overexpressed on tumor cells, increasing the tumor accumulation of the Ini@PM-HP. The intratumorally released iniparib acted as the chemotherapy drug to promote the cell apoptosis by targeting PARP and dysfunctioning the DNA damage repair mechanism. Meanwhile, the oxygen can be generated from the reaction between the Mn chelated with the tetrakis(4-carboxyphenyl) porphyrin (named as Mn-TCPP) and the H_2_O_2_. Accompanied with the irradiation by the 808 nm and 650 nm laser, the combined PDT/PTT/chemotherapy can be performed on the cancer cells or tumor tissue. The *in vitro* results indicated that after the combination therapy, the cell viability of MDA-MB-231 is the lowest, with 88% inhibition rate, showing the best therapeutic effect. The *in vivo* results further demonstrated that the combination therapy group achieved the comprehensively suppressed effect on the tumor growth, with the lowest relative tumor volume [250]. The H&E staining and immune-histochemical analysis of the treated tumors results demonstrated that compared with other groups, the most apoptotic cells and weakest cell proliferation signal existed in combinatory therapy group sample. Furthermore, the immune-histochemical analysis (γ-H2AX, a DNA damage and repair marker) showed that after the combinatory treatment, the downregulation of the HIF-1α, which was caused by the hypoxia alleviation and the PDT, could further enhance DNA damage by stimulating the degradation of PARP-1, promoting the chemotherapy drug efficiency.

To achieve the purpose of simultaneous synergistic treatment efficacy, Cheng et al. reported multi-modal therapeutic NPs with tumor targeting ability, named IR&DOX@NC, which was composed of the GNR, mesoporous organsilica, loaded with new indocyanine green (IR820) and DOX and the coated by HA. After the 4T1 cancer cells uptake, the intracellular hyaluronidase and GSH can degrade the HA and organosilica, dual-triggering the release of the loaded DOX and IR820. With the 808 nm laser irradiation, PTT/PDT/chemotherapy triple-combination can be simultaneously achieved because of the GNR, released IR820 and DOX. The *in vitro* results showed that after the combination treatment, the 4T1 cell viability was lower than 5%, and the *in vivo* anti-tumor results demonstrated that compared with other groups, the IR&DOX@NC with laser exhibited the best tumor growth suppression effect, with treatment effect of complete tumor eradiation on three mice after five times treatments (five times NPs injection and laser irradiation) (Fig. 5C). Furthermore, the terminal deoxynucleotidyl transferase (TdT)-mediated dUTP (2′-Deoxyuridine and 5′-Triphosphate) nick-end labeling assay results on the tumor regions showed that much higher amount of apoptotic cells were found in combination therapy group samples, which were in good accordance with the western blots results demonstrating remarkable increase of the downstream protein active-caspase 3 in combination therapy group [251].

More representative NPs-based PTT, PDT studies combined with chemotherapy are presented in Table 5.

**Table 5:** NP-based combined PTT/PDT/chemotherapy.^a^

The NPs	PT agent	PSs	Chemotherapy drug	*In vitro* anti-cancer effect	Particles injection dosage of *in vivo* anti-cancer study	*In vivo* anti-cancer effect	Reference
GNRs-MPH-^ALA/DOX^-PEG	GNR	5-ALA	DOX	MCF-7 cell viability: less than 40%	200 μL (Au: 200 μg, DOX: 21 μg, ALA: 32 μg)	The MCF-7 tumor almost completely disappeared	[252]
DOX@BPNs@MnO2	BPNs	BPNs	DOX	Hela cell viability: less than 15%	150 μL (4.5 mg/kg)	The Hela tumor was almost completely suppressed without obvious recurrence during this therapeutic process	[253]
BDP-T-N&DTX@PS-g-PEG-FA	BDP-T-N	BDP-T-N	DTX	4T1 cell viability: less than 15%	6 mg/kg	exhibited highest antitumor efficacy, the relative 4T1 tumor volume close to 0	[254]
Fe_3_O_4_@GO@Ce6@ mucin 1 aptamer-PTX	Fe_3_O_4_ and graphene oxide	Ce6	PTX	MCF-7 cell viability: less than 20%	*N/A*	*N/A*	[255]
DOX&ALS@ TRP-PEI-PEG-FA micelles	ALS	ALS	DOX	Hela cell viability: less than 10%; A549 cell viability: less than 40%	1 mg/kg (equivalent ALS content)	∼97% antitumor efficiency on HeLa xenografted tumors	[256]
IR780-Biotin/Quercetin	IR780	IR780	Quercetin	4T1 cell viability: approximately 20%;	2 mg/kg (equivalent quercetin content or IR780 content)	the 4T1 tumor inhibition ratios: 96%	[257]
AuNR@Porphyrinic MOFs@CPT	AuNR	porphyrinic MOFs	camptothecin	4T1 cell viability: 12%	3.5 mg/kg (equivalent TCPP content)	The 4T1 tumor growth was remarkably suppressed, with the smallest tumor sizes	[258]
TPZ@CaCO_3_@PDA-ICG-TPGS-RGD	PDA	ICG	TPZ	U87MG The IC_50_ values in normoxia were 0.15 μg/mL (ICG concentration) and The IC50 values in hypoxia were 0.08 μg/mL, much lower than those in normoxia	1.5 mg/kg (equivalent ICG content)	Inhibition rate of the U87MG tumor was ∼87%	[259]

^a^ The undefined abbreviations in the table: polyethyleneimine (PEI), OHC-PEG-CHO and PEI (PP), docetaxel (DTX), 2-ethynylthiophene and 4-(dimethylamino)benzaldehyde modified boron dipyrromethene (BDP-T-N), amphiphilic poly(styrene-co-chloromethyl styrene)-graft-poly(ethylene glycol) (PS-g-PEG), 5- aminolevulinic acid (5-ALA), P(AAm-co-AN) (TRP), tirapazamine (TPZ), D-α-tocopheryl PEG 1000 succinate (TPGS), one synthesized-photosensitizer (named as ALS).

### 3.3 PTT, PDT combined with immunotherapy

The hyperthermia from PTT treatment or ROS from PDT treatment can induce immunogenic cell death of cancer cells, with the release of adjuvant-like danger signals as well as cancer specific adjuvants [118,260,261]. It is therefore meaningful to investigate the combination of PTT/PDT and other immunotherapeutic agents, like immune checkpoint inhibitors, to achieve the best immunological activation against cancer and potentiate the abscopal effect of the treatment [96]. PTT and PDT treatments are amongst the treatments that induce immunogenic cancer cell death, leading to priming and activation of the immune system against the tumor [134,[260], [261], [262], [263], [264]].

**Fig. 6:**
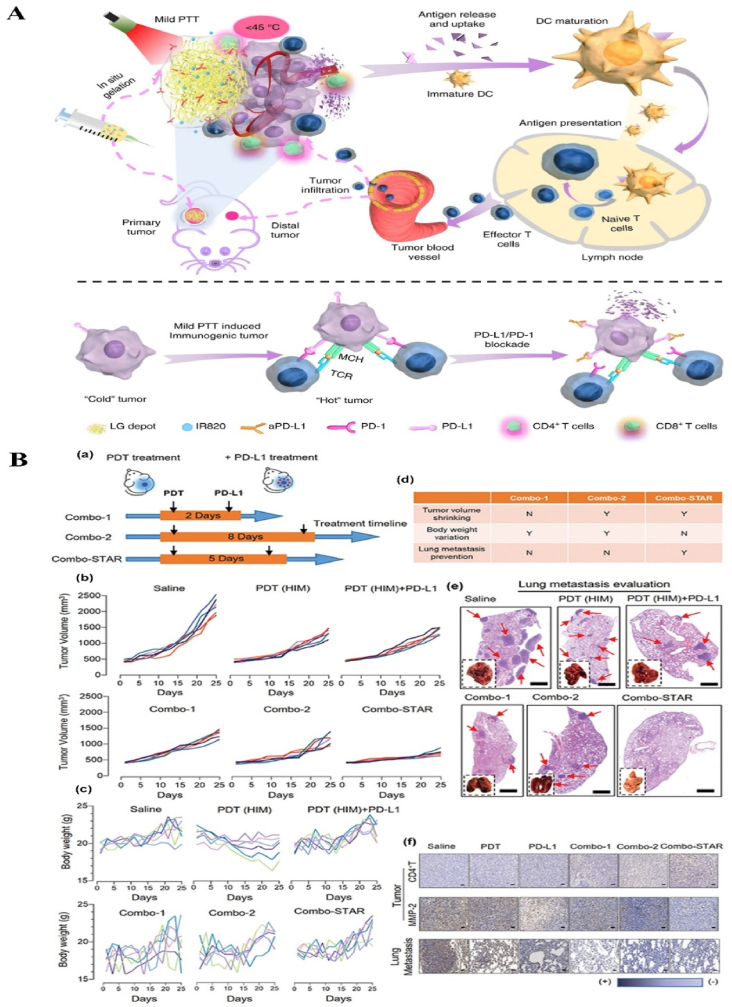
**NP-based innovative combined PTT/PDT/immunotherapy nanosystems. A. Scheme illustrating the immunological effect of mild PTT combined with the release of immune checkpoint inhibitor.** Adapted from Ref. [266]. Copyright *©* 2019, Springer Nature. **B. Analysis of the best time interval between PDT and administration of immune checkpoint inhibitor on tumor growth and metastasis development in 4T1 model**: (a)schematic of the different treatment regimens; (b) *in vivo* antitumor efficacy of the combined PDT/immunotherapy (n = 6); (c) variations in body weight for the different treatment groups for the duration of the study; (d) summary of overall efficacy and safety of the different treatment regimens; (e)H&E staining of pulmonary tissues to highlight the presence of metastases after treatment (scale bar 1000 μm; metastases highlighted by red arrows); (f) images of tumors and lungs stained for CD4^+^Tcells and for cancer metastasis biomarker (MMP-2) (scale bar 50 μm). Adapted from Ref. [267]. Copyright *©* 2022, Wiley-VCH.

The localization of the NPs (extra *vs.* intracellularly; endoplasmic reticulum (ER), mitochondrial level) and the intensity of the PTT/PDT treatment (laser intensity) are variables influencing the mechanisms of immunogenic cell death and the magnitude of immune activation [27,29,127,265,266]. Targeting the NPs to the ER *via* pardaxin peptides increased the levels of ER stress and the exposure of calreticulin on the surface of the cells after NIR irradiation. This increase in the fraction of danger associated molecular patterns elevated the fraction of CD8^+^ T cells, while decreasing the number of regulatory T cells. Pro-inflammatory cytokines are increased, while immunosuppressive cytokines are decreased [29]. A mild, controlled hypothermia (lower than 45 °C) sensitized immunologically “cold” tumors to the action of immune checkpoint inhibitors, providing the best results of the combination (Fig. 6A) [266].

To better boost the anticancer immunity for the combined PDT/immunotherapy, Zhang et al. fabricated a semiconducting polymer-poly(cyclopentadithiophene-alt-benzothiadiazole) (PCB)-based NPs (named as SPN_pro_), which conjugated with the proteolysis targeting chimera *via* a Cathepsin B cleavable linker. The proteolysis targeting chimera includes an Indoleamine 2,3-dioxygenase inhibitor and an E3 ligase-biding peptide. The experiments results indicate that after the 808 nm laser irradiation, the intratumorally accumulated NPs can generate ^1^O_2_ due to the PCB part and then the PDT-induced immunogenic cells death further activated the anti-cancer immune response. Meanwhile, the tumor-overexpressed Cathepsin B released the proteolysis targeting chimera for the Indoleamine 2,3-dioxygenase-targeting proteolysis and degradation, alleviating the tryptophan consumption and kynurenine accumulation and finally reversing the immunosuppression. The combination of the anticancer immune activation and immunometabolism intervention led to the strongest T cells immune response when compared with other groups. The *in vitro* results show that at the PCB concentration of 40 μg/mL, after 6 min laser irradiation, only 10% of 4T1 cells survived compared with control group. The *in vivo* results showed that only in combined PDT/immunotherapy group, the distant tumors were greatly inhibited and primary tumor were completed suppressed at the same time [264].

The treatment schedule between laser irradiation, PDT and administration of immune checkpoint inhibitors should be optimized based on the molecular mechanisms of the immune activation promoted by PDT. Wu et al. investigate the optimal schedule by inducing immunogenic cell death with HA-based NPs loaded with ICG and administered the immune checkpoint inhibitor at 0, 2, 5, or 8 days after the laser irradiation [267]. Only the treatment schedule with 5 days interval between the laser irradiation and the administration of the immune checkpoint inhibitor can control the 4T1 tumor growth and prevent the cancer metastasis (Fig. 6B). Furthermore, the authors also employed 3D stimulated Emission Deletion imaging with super resolution to visualize the interaction between T cells and cancer cells in the optimal treatment schedule. The three stages in the process of the enhanced immune blockade efficacy in optimal group were clearly observed [267].

The entity of the immune response induced by the PTT or PDT effect can be increased by loading adjuvant molecules (e.g., imiquimod) within the particles. Jiang et al. developed a MoS_2_–CuO-based hetero-nanocomposites for the synergetic PTT/chemodynamic/immunotherapy for cancer treatment. With the 808 nm laser irradiation, MoS_2_ increased the tumor temperature, promoting cancer cell necrosis with a photothermal conversion efficiency of ∼25%. At the same time, Cu ions catalyzed the formation of intracellular hydroxyl radicals leading to cancer cell apoptosis. The release of tumor-associated antigens from the dying cancer cells was intercepted by antigen presenting cells which has been activated by the imiquimod released from the particles. Both *in vitro* and *in vivo* indicated the increased expression of co-stimulatory signals CD80 and CD86 after the laser irradiation, demonstrating the synergistic anti-tumor effect in CT26 colon cancer model. The *in vivo* results showed that after intratumorally injecting the NPs into the primary tumor, followed by 808 nm laser irradiation, the treated mice survived longer than all the other groups, with 65% of the animals still alive after 45 days and a controlled tumor growth in both primary and secondary tumor up to 16 days post treatment mirrored by an increase in CD4^+^ and CD8^+^ T cells in the spleen [268].

Recent representative NPs-based PTT, PDT studies combined with immunotherapy are presented in Table 6.

**Table 6:** NP-based combined PTT/PDT/immunotherapy.

The type of NPs	Payload	PTT/PDT	*In vitro* anti-cancer effect	Particles injection dosage of *in vivo* anti-cancer study	*In vivo* anti-cancer effect	Reference
Au NPs grown *in situ* over PSiNPs	Tumor associated antigens present on cell membrane	PTT	Immunostimulation of antigen presenting cells	50 μL (1 mg/mL)	Combination with immune checkpoint inhibitor reduces tumor growth in 4T1 tumor model	[96]
Melanin NPs coated with cancer cell membrane	Tumor associated antigens present on cell membrane	PTT	Increased expression of calreticulin on 4T1 cells irradiated with laser	100 μL (4 mg/mL) for PAI, no exact dose information for the PTT *in vivo* study	Combination PTT and indoleamine 2,3-dioxygenase inhibitor reduces tumor growth in 4T1 tumor model	[269]
Melanin NPs coated with a silica shell	azodiisobutylimidazoline hydrochloride	NIR II window PTT and generation of free radicals upon release of the payload	Mitochondrial toxicity in 4T1 cells	200 μL (100 μg)	Combination with *anti*-PD1 reduced tumor burden in established 4T1 tumors. Repolarization of M2-like macrophages to M1. Reduction in regulatory T cells in the distal tumors.	[127]
PDA NPs coated with macrophage cell membrane	TMP 195, a compound with macrophage repolarization ability	PTT and PTT-mediated release of TMP 195	Repolarization of Raw 264.7 macrophages from M2 to M1. Irradiation of the particles reduces the efficacy of repolarization	10 mg/kg (equivalent PDA dosage)	Hyperthermia (about 50 °C). Control over tumor growth for 15 days. Reduction in regulatory T cells in the tumor. Reduction in the fraction of myeloid derived stem cells and increase in M1-like macrophages, correlate with a total increase of tumor-associated macrophages.	[126]
Positron-guided MSN	CpG oligonucleotide as antigen, Ce6 as PSs,^64^Cu as tracer for positron emission tomography, neoantigens peptides conjugated on the surface of the particles	PDT	No increased activation of DCs after laser irradiation compared to particles alone. Overall increase in the activation of DCs.	At the equivalent dosage of 20 μg CPG, 30 μg Ce6, 19 μg neoantigen peptide of MC-38 tumor (Adpgk) and 120 μg biodegradable MSNs	Control in primary and distal tumor growth in M38 and B16.F10 models. Activation of DCs and T cells.	[270]
Cationic organic frameworks composed of PEI and porphyrin	shPD-L1 for inhibition of PD-L1 expression in tumor cells	PDT, fluorescence and PAI	Dose-dependent toxicity in 4T1 cells after laser irradiation. High transfection levels with reduction in the expression of PD-L1 on the surface of 4T1 cells after 48 h	10 mg/kg (equivalent cationic flexible organic framework NPs)	Tumor accumulation after intravenous administration. Control over tumor growth in 4T1 tumor model. Reduction in the expression of PD-L1 and increase in both CD4^+^ and CD8^+^ T cells in the tumor	[271]
MOFs based on zirconium ions and H_2_TCPP	Acriflavine as antihypoxic molecule and CpG oligonucleotide as adjuvant	PDT	Increased production of ROS and antihypoxia signaling in H22 cells. Increased maturation of DCs	10 mg/kg	Control over tumor growth in H22 model. Reduction in the expression of matrix metallo proteinases 9, vascular endothelial growth factor. Increased percentage of mature DCs	[272]
SPN_pro_ based on the semiconducting polymer PCB	proteolysis targeting chimeras	PDT, NIR fluorescence	4T1 cancer cell viability decreased to around 10%	200 μL (equivalent PCB concentration: 200 μg/mL)	The primary 4T1 tumors were completely suppressed and the distant tumors were also greatly suppressed	[264]

### 3.4 PTT, PDT combined with RT

**Fig. 7:**
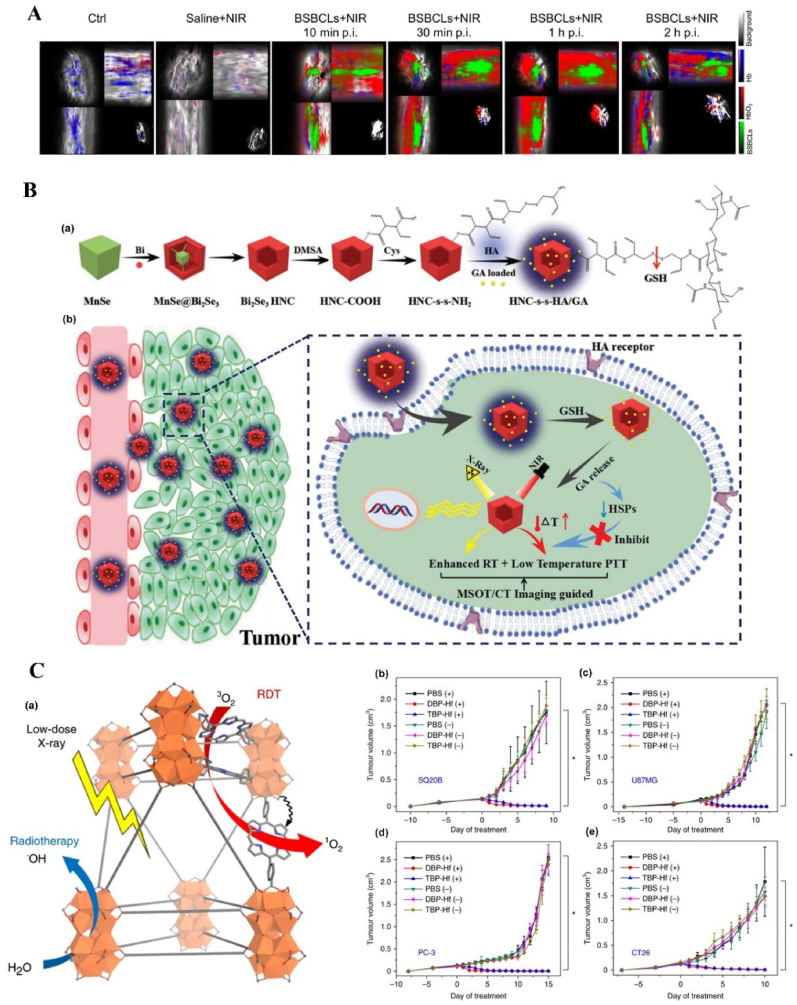
**NPs-based innovative combined PTT/PDT/RT nanosystems. A. PAI of the tumor site after injection with BSBCLs nanosystems as a function of time.** Adapted from Ref. [277]. Copyright *©* 2021, American Chemical Society. **B. Illustration of synthesis procedure of Bi**_**2**_**Se**_**3**_**HNC‐s‐s‐HA/GA (HNC‐s‐s‐HA/GA) NPs and HNC‐s‐s‐HA/GA induced combined low‐temperature PTT and enhanced RT.** Adapted from Ref. [288]. Copyright *©* 2019, Wiley-VCH. **C.** (a) schematic illustration of the mechanisms of X-ray-induced RT and ^1^O_2_ generation; (b) tumour growth curves of SQ20B, U87MG, PC-3 and CT26. Tumour-bearing mice treated with PBS, DBP-Hf or TBP-Hf, with (+) or without (−) X-ray irradiation (*n* = 6). Adapted from Ref. [306]. Copyright *©* 2018, Springer Nature.

Recently, the combination of RT with PTT has become an attractive strategy for cancer diagnosis and treatment [273]. The hyperthermia induced by PTT accelerates the local blood flow [274], which helps to alleviate hypoxia-associated radio-resistance [275,276]. A recent paper published by Xiang et al. recorded the oxygen level in TME by PAI after intratumorally injecting their bismuth-based nanosystem (named as BSBCL) [277]. After a 10-min PTT and a local temperature increase to 47.2 °C, it was found that the hypoxia was significantly relieved (Fig. 7A). Oxygenated hemoglobin was visible after 10 min of NIR laser irradiation and perfumed the tumor site after 1 h. Even after 2 h, the oxygenated hemoglobin was still presented, while in control only deoxygenated hemoglobin was identified. *In vivo* results showed that the heat, which was generated by PTT, combined with the DNA cleavage effects from X-ray irradiation had been proved to have synergistic effects on tumor ablation [[278], [279], [280], [281], [282], [283], [284], [285], [286], [287]].

In addition to the enhanced therapeutic effects, the combination of hyperthermia and RT enables lower NIR-laser power and/or less X-ray doses, thus decreasing the damaging side effects on the normal adjacent tissues. Song et al. reported a tumor-targeting nanoplatform for synergistic low-temperature photothermal RT (Fig. 7B). The nanoplatform is based on bismuth selenide (Bi_2_Se_3_) hollow nanocubes, which acts a dual role as a photothermal agent and a radiosensitizer. To increase the stability, biocompatibility and targeting capacity, the Bi_2_Se_3_ hollow nanocubes were modified with HA *via* a redox-cleavable disulfide bond. The nanocubes were further loaded with gambogic acid, a HSP inhibitor. After X-Ray and NIR laser irradiation, the HSP inhibitor was released to combat the heat-stress resistance, to enhance the PTT effects albeit a mild hyperthermia (about 45 °C) at tumor site. The combination with RT efficiently controlled 4T1 tumor growth with only one low-power 808 nm laser exposure (0.5 W/cm^2^) and X-ray irradiation(6 Gy), and showed superior efficacy compared with either RT or PTT alone [288].

Similar to PTT, the combination of PDT with RT emerges as a promising synergistic cancer treatment approach [43,[289], [290], [291], [292], [293], [294], [295]]. There are two main categories of PDT/RT combinations, depending on whether NIR irradiation is needed. One strategy requires sequential NIR and X-ray irradiation, similar to those PTT/RT combinational strategies discussed in the previous paragraphs. For example, Qiao et al. designed an innovative oxygen-generating microalgae for PDT/RT. The live algae was simply camouflaged by RBCM and generated oxygen by photosynthesis upon exposure to the red light. The oxygen generated enhanced the radiotherapeutic efficacy, and the X-ray irradiation in turn killed the algae and release the intracellular chlorophyll, acting as PDT PSs. The bioengineered microalgae, after sequential exposure to red light with the generation of oxygen, X-ray as RT to release chlorophyll, and laser for PDT, led to complete 4T1 tumor elimination in the intratumorally injected mice, and successful tumor suppression in the mice by intravenous injection [296].

Another strategy, which is also known as radiodynamic therapy, simply relies on X-ray irradiation [[297], [298], [299]]. An energy transducer (mostly scintillator) is first exposed to X-ray to emit optical luminescence, which excites PSs to generate ^1^O_2_ [300], paving the way to circumvent relatively restricted photon penetration in PDT [301]. Compared with traditional RT, radiodynamic therapy is expected to achieve therapeutic outcomes with lower X-ray doses [[302], [303], [304], [305]]. For example, Lu et al. designed two nanoscale MOFs (nMOFs), based on hafnium (Hf) clusters working as X-ray scintillators and porphyrin ligands as PSs as shown in Fig. 7C. A single intratumoral injection of 5,15-di(*p*-benzoato)porphyrin-Hf (DBP-Hf), followed by ultra-low X-ray irradiation (0.5 Gy, 3 daily doses), eliminated tumor efficiently on all the tumor models. The *in vivo* results showed that the control MOF based on zirconium (DBP-Zr) did not show any anticancer efficacy, but the intravenous administration of DBP-Hf with low-dose X-ray irradiation exhibited inhibited tumor growth, though not tumor elimination [306].

Some representative NPs-based PTT, PDT studies combined with RT are presented in Table 7.

**Table 7:** NP-based combined PTT/PDT/RT.

Type of NPs	Payload	PTT/PDT	*In vitro* anti-cancer effect	Particles injection dosage of *in vivo* anti-cancer study	*In vivo* anti-cancer effect	Reference
Silica-coated bismuth NPs	H_2_O_2_-responsive N-benzylaminoferrocene-based prodrug	PTT	Photo- and H_2_O_2_-induced GSH-depletion and apoptosis	50 μL (in terms of bismuth NPs: 2 mg/mL)	PTT-induced hypoxia alleviation. Sequential PTT and RT inhibited 4T1 tumor growth	[277]
Liposomal iridium nanocrystals	*N/A*	PTT	RT-induced DNA damage on 4T1 cells	6 mg/kg (equivalent Iridium content)	PAI, and inhibited 4T1 tumor growth by RT + PTT	[278]
2D silicene nanosheets decorated by Pt and lipids	*N/A*	PTT	Oxygenation of 4T1 cells catalyzed by Pt. Combined PTT and RT induced cell death.	15 mg/kg	4T1 tumor hypoxia alleviation and tumor growth inhibition	[281]
Cu_3_BiSe_3_ NPs modified by poly(vinylpyrollidone)	*N/A*	PTT	ROS generation and Hela cell killing effects	25 μL (4 mg/mL)	Inhibited Hela tumor growth and metastasis	[282]
Bi_2_Se_3_ hollow nanocubes modified by HA	Gambogic acid	PTT	4T1 cell HSP expression inhibition after PTT, and cell killing effects	100 μL (5 mg/mL)	4T1 tumor inhibition by mild hyperthermia (∼45 °C) + RT	[288]
PEG-modified nanoscintillator composed of Gd2(WO4)3:Tb NPs	Merocyanine 540	PDT	Apoptosis of 4T1	30 μL (7.5 mg/mL)	Dual-modal CT/MRI imaging and 4T1 tumor growth inhibition	[292]
Lipids coated Hf-incorporated AIE photosensitizer NP	*N/A*	PDT	Bioorthogonal coupling of NPs on 4T1 cell membrane followed by X-ray irradiation	25 μL (tetraacetylated N-azidoacetylmannosamine: 0.08 mg)+ 6 mg/kg (equivalent Hf content)	*In vivo* targeting by bioorthogonal click chemistry and 4T1 tumor growth inhibition	[294]
RBCM-coated Algae	Chlorophyll	PDT	Intracellular oxygen level increase and 4T1 cell killing by RT + laser irradiation	150 μL (1 × 10^6^ RBCM-Algae/mL)	4T1 tumor inhibition by ROS generation. HIF-α and VEGF expression decrease.	[296]
MOFs, based containing Hf or Ru	*N/A*	PDT	Mitochondria targeting and apoptosis of MC38 cells	0.2 μmol (equivalent dose of [DBB-Ru bis (2,2′-bipyridine)(5,5′-di(4-benzoato)-2,2′-bipyridine)Ru (II) chloride])	MC38 tumor growth inhibition	[302]
MOFs, based on Hf clusters and porphyrin ligands	A small-molecule IDO inhibitor	PDT	Intracellular ^1^O_2_ generation and cell growth inhibition	0.11 mg	Ultra-low X-ray irradiation (0.5 Gy, 3 daily doses), eliminated tumor efficiently on CT26, U87MG and TUBO models	[306]

### 3.5 PTT, PDT combined with other cancer therapies

In addition to chemo-, immuno- and RT, there are other options combined with phototherapies, such surgical therapy and gene therapy. A brief introduction of each of these combinational therapies, together with prototype applications, can be found below.

Surgery is the most widely used in clinical cancer therapy to remove the primary tumor, but usually not potent enough to eliminate tumor residuals, which usually lead to recurrence and metastasis [307]. Thus, traditional surgery is usually followed by systemic chemotherapy [308]. In other cases especially breast cancer, chemotherapy is applied before surgery to shrink the tumor and make the margin clearer for the surgery [309]. For both applications, PTT and PDT can be introduced as adjuvant therapy to surgery [310,311]. Specifically, PTT could be applied during the surgery right after the tumor excision, to eliminate residual tumor and prevent bacterial infection after surgery. Otherwise, PTT or PDT can be applied after the surgery to suppress the recurrence and elongate survival [[312], [313], [314]]. Moreover, when combined with immune checkpoint inhibitors, surgery followed by PTT or PDT demonstrated great potential for both primary and metastatic tumor treatment [315,316].

**Fig. 8:**
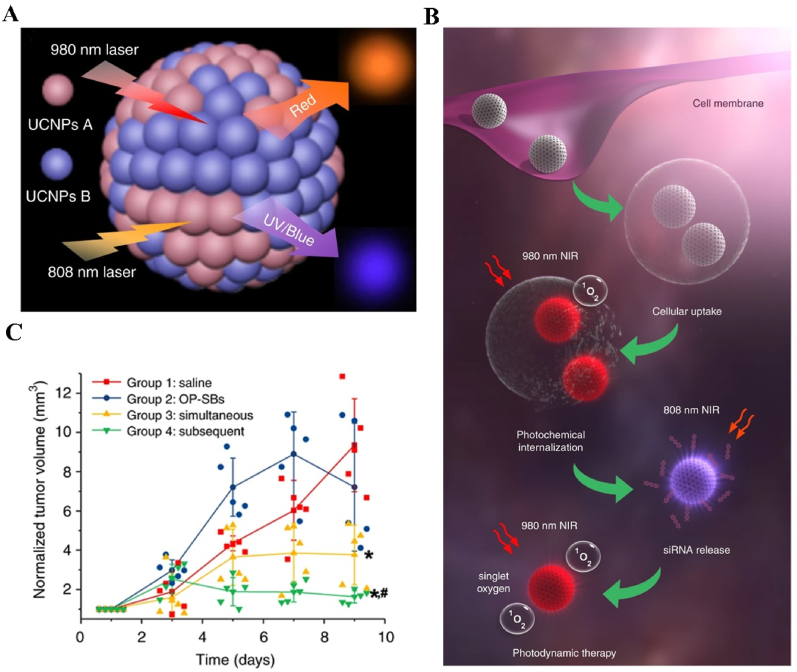
**NP-based innovative combined PTT/PDT/gene therapy nanosystems. A. Scheme of photo-programmable excitation of mixed UCNPs. B. Schematic illustration of orthogonal excitation of PSs and azobenzene-based caps for endosomal escape, siRNA release and PDT. C. Cal27 tumors growth curve on Balb/c nude mice injected with different nanoformulations and irradiated with a 980 and/or 808 nm NIR laser.** Adapted from Ref. [326]. Copyright *©* 2019, Springer Nature.

Gene therapy refers to the delivery of genetic materials (DNA, RNA, oligonucleotides, Clustered Regularly Interspaced Short Palindromic Repeats (CRISPR), etc.) to kill tumor cells or alter the abnormal TME [317]. The combination of gene therapy and PT has long been investigated [16,[318], [319], [320], [321], [322]]. On the one hand, gene delivery and expression benefits from the hyperthermia from PTT [319], or the ROS-induced lipid membrane disruption from PDT (also known as photochemical internalization) [323]. On the other hand, the limited efficacy of PTT and PDT could be mitigated by gene expression modulation. For example, HSP overexpression facilitates cancer cell survival after hyperthermia [324], and HIF-1a overexpression induced hypoxic environment limits the efficacy of PDT [325]. Thus, gene therapy and PT can complement each other to achieve better therapeutic outcomes. For example, Zhang et al. reported a programmable photo-activated gene/PDT combinational therapy strategy using modularly assembled UCNPs (Fig. 8A). One UCNP in the nanoassembly converted 908 nm laser to red emissions, which triggered ^1^O_2_ production by the encapsulated PS. The other UCNP in the nanoassembly converted 808 nm laser to UV/Vis emissions, which cleaved the UV-sensitive azobenzene to release the encapsulated small interfering RNA (siRNA). The siRNA was expected to knockdown superoxide dismutase 1 (SOD1) which is responsible to scavenge free radicals and restrict PDT. The *in vivo* results indicated that the sequential NIR irradiations led to sequential photochemical internalization, siRNA cytoplasmic delivery and PDT led to tumor growth suppression after intratumoral administration, as shown in Fig. 8B and C [326]. This proof-of-concept study demonstrates the potential to apply gene and PT synergistically.

Some representative NPs-based PTT, PDT studies combined with other cancer therapies are presented in Table 8.

**Table 8:** NP-based combined PTT/PDT/other therapies.

The type of NPs	Payload	PTT/PDT	*In vitro* anti-cancer effect	Particles injection dosage of *in vivo* anti-cancer study	*In vivo* anti-cancer effect	Reference
Au nanoshells conjugated with ICG	*N/A*	PTT with surgery	Photothermal killing of SGC-7901 cells	100 μL (200 μg/mL)	Inhibited tumor growth in subcutaneous SGC-7901 gastric tumor and peritoneal metastasis model (after image-guided surgery)	[312]
Prostate-specific membrane antigen–targeted PDT agent	*N/A*	PDT with surgery	*N/A*	0.5 mg/kg	Primary and metastasis PC3 tumor targeting. Combined PDT with image-guided surgery reduce tumor recurrence.	[313]
AIE organic NPs	*N/A*	PDT with surgery	PDT induced 4T1 cell killing	100 μL (800 μM)	PAI guided surgery combined with PDT inhibited tumor growth	[314]
A tumor-penetrating peptide based hydrogel	JQ1 (bromodomain and extraterminal protein BRD4 inhibitor) and ICG co-loaded tumor cells	PDT with surgery	DCs maturation	2.5 mg/kg (equivalent ICG content)	DCs maturation and T cell proliferation. Inhibited the postoperation 4T1 tumor recurrence and metastasis	[315]
Polymer nanocomplex composed of PEI and copolymer of PEG, histidine and glutamic acid	Plasmid DNA of p53 and KillerRed	PDT with gene therapy	Tumor pH-triggered expression of p53 and KillerRed in H1299 cells; apoptosis after laser irradiation	100 μL (10 μg pCMV-p53 and 10 μg pKillerRed-mem)	p53 and KillerRed expression in an aggressive H1299 mouse-tumor model and tumor inhibition by either intratumoral or intravenous injection	[320]
PDA-coated nucleic acid nanogel	HSP 70 siRNA	PTT with gene therapy	Knockdown of HSP70 and apoptosis of Hela cells	200 μL (equivalent siRNA content: 1 mg/kg)	Low power PTT induced Hela tumor growth	[321]
Assembled upconversion NPs	SOD1 siRNA and the PSs (Zinc phthalocyanine)	PDT with gene therapy	SOD1 knockdown in HeLa and Cal27 cells, followed by PDT to kill cells	50 mg/kg	Cal27 tumor growth inhibition after intratumoral administration	[326]

## 4 The clinical transformation potential of the NPs-based PT system

PTT and PDT are considered as favorable approaches among several cancer treatments due to their non-invasive characteristic and their very promising therapeutic potential. Nonetheless, despite the significant preclinical interests demonstrated by the number of studies carried out in the last 15 years, their clinical translation is still preliminary [7,63,327,328]. In this section, we discuss the various limitations that the current PTT and PDT-based treatments face and suggest certain ways to overcome these limitations, and we summarize the various nanosystem-based treatments in clinical trials.

### 4.1 The clinical potential of NPs-based PTT

The large number of preclinical studies on PTT-based therapeutics in the last 15 years demonstrates the increased scientific interests and their potential in cancer therapy. Unfortunately, despite the promising results of several of these studies, the translation of the various PTT-developed therapeutics into the clinical stage is still hindered by the significant limitations that these systems present. As previously described, PTT can kill the cancer cells by increasing temperature after irradiation with a light source, usually an NIR laser (700–1700 nm). Although this method could be very useful for specific cancer types like melanoma (skin cancer), it cannot be used in tumors found in deep tissues or close to large blood vessels. In the former case, the limitation derives from the fact that the commonly used NIR lasers (700–1000 nm) cannot penetrate tissues with a thickness of more than a few centimeters [329]. In the latter case, lack of PTT agents can lead to heat dissipation due to the ‘heat sink’ effect [330,331], reducing the therapeutic effect in the cancer tissue. It should be noted that this dissipation could damage the surrounding tissues, especially if high-intensity lasers are used. One of the ways to overcome the thickness limitation is to use multiple interstitial fibers for tumors located deep in the body [332].

To overcome the above limitations and to enhance the PTT effect, several photothermal agents, from small molecules like ICG to various nanostructures, such as the metallic NPs like Au, graphene, QDs, hybrid-like Au-coated silica (ClinicalTrials.gov:*NCT* 04240639, Table 9), transition metal dichalcogenides) [328] can be used. Although these agents improve the PTT efficacy, they are also governed by their limitations, impeding the PTT's clinical translation. A few of the major limitations include the low tumor accumulation, their non-controlled biodistribution, and the low PTT efficacy. In the case of small molecules, the low accumulation could be controlled by the local administration of the agent at the tumor site. Nevertheless, local administration is not always feasible, and if it is, it may increase the agents' concentration peritumorally but not inside the tumor due to the poor diffusion of the injected agent. Various NPs can be used either individually or in combination with the already used small molecules, to solve this problem. The advantage of using NPs is that as mentioned before, their surface could be easily functionalized or modified, enhancing the tissue specificity and improving their bio-distribution. NPs can also improve the photothermal conversion efficacy that some small molecules like ICG, melanin, or phthalocyanine molecules demonstrate [91,[333], [334], [335], [336]]. Additionally, using NIR-II (1000–1700 nm) and PTT agents with a high extinction coefficient in this wavelength could also improve the overall efficacy of the used systems [337].

**Table 9:** Nanosystem-based PTT/PDT clinical trials in the last 10 years.

Clinical Trials Types	Condition	Intervention/Treatment/Drug	Status	ClinicalTrials.gov Identifier
PTT	Head and Neck Cancer	AuroLase Therapy (silica core coated surrounded by an Au shell)	Completed: August 2014	*NCT*00848042
			Terminated: June 2014	*NCT*01679470
			Completed: October 2020	*NCT*02680535
	Neoplasms of the Prostate	AuroShell (silica core coated surrounded by an Au shell) particle infusion	To be completed: June 2023	*NCT*04240639
PDT	Actinic Keratosis	BF-200 ALA gel (nanoemulsion)	Completed: April 2015	*NCT*01966120
	PDT Microvesicle Particle	Cream containing 4% Imipramine	To be completed: September 2022	*NCT*03960125
	Neoplasms, Basal Cell Carcinoma, Basal Cell Photochemotherapy Photosensitizing Agents	Hexylaminolevulinate cream, Aminolevulinic Acid Nano Emulsion & Methylaminolevulinate cream	To be completed: December 2025	*NCT*02367547
	Blastic Plasmacytoid Dendritic Cell Neoplasm Acute Myeloid Leukemia Acute Lymphocytic Leukemia Myeloproliferative Neoplasm	IMGN632 injection (Targeted antibody-drug conjugate)	To be completed: December 2022	*NCT*03386513

NPs have shown great potential for several medical applications including PTT. However, there are still unanswered questions concerning their biosafety in the short or long term, especially regarding QDs, inorganic NPs represented by Au, Cu, and hybrid NPs, such as Au-iron oxide. While these NPs can significantly improve the PTT effect, the lack of clinical investigations regarding their long-lasting effects impedes their robust use. Inorganic NPs could be replaced with organic biodegradable ones, reducing their potential toxicity. Nevertheless, organic NPs are not as efficient as their inorganic counterparts.

Another limitation that also needs to be considered is the heating resistance of the cancerous tissues. This enhanced heat-shock response demands either high laser intensities, increased PTT agent concentration, or high photothermal conversion efficiency, imposing one more impediment to the use of clinical use of the current systems. To overcome this limitation, PTT could be combined with other therapeutic approaches like chemo-, immune- and RT [7,327], resulting in enhanced therapeutic outcomes.

It is evident from the above that many obstacles need to be solved before translating the NPs-based PTT systems into clinics. This is further supported by the number of clinical trials (Table 9) performed in the last ten years. Despite the significant advances, only one system (AuroShell) which has been introduced above is currently under clinical trials. The latest study is scheduled to be completed in 2023.

### 4.2 The clinical potential of NPs-based PDT

PDT is an alternative form of cancer therapy that uses a light source and the ROS generator-PSs. In contrast to PTT, the photoactive agent does not increase its temperature due to laser irradiation but transfers energy to the oxygen molecules of the surrounding tissue [7,63], subsequently leading to the overproduction of ROS, and then resulting in cancer cells’ death. The common ground between PTT and PDT is using a light source and the photoactive agent, rendering PDT to the same limitations as the ones described in the PTT section above [7,63]. Thus, overcoming the limitations mentioned above can improve the therapeutic efficacy of NPs-based PDT systems.

One approach to overcome the depth penetration limitation could be using luminescence proteins and NPs or QDs, creating self-illuminating systems, which has been introduced above too, but unfortunately have not been extensively studied [[338], [339], [340]].

Additionally, it should be noted that PDT is not limited by the heat resistance of cancer cells since its mechanism of action lies in the generation of ROS. Nevertheless, the same mechanism acts also as a hurdle to its clinical translation due to the hypoxic nature of the cancerous tissues. The low amount of oxygen in the cancerous microenvironment imposes a significant barrier to an effective PDT treatment since it does not allow for a significant generation of ROS [63]. Amelioration of tumor hypoxia through oxygen generation or reduction of oxygen consumption could be potential solutions to overcome this limitation [341]. Moreover, the use of agents in the form of small molecules or NPs that promote the Fenton reaction (formation of hydroxide (OH^−^) and·OH) [342] or hypoxia-responsive therapeutics could also enhance the PDT effect. As in PTT, an improved outcome could also be achieved by using PDT in combination with one or more of the existing cancer therapies.

At this stage, an Aminolevulinic Acid Nanoemulsion-based PDT clinical trial (ClinicalTrials.gov:*NCT02367547*) for basal cell carcinoma conducted by three Finish authorities, including Tampere University and University of Jyväskylä, is ongoing.

To date, besides the trial mentioned above, as far as we know, only other four nanosystems-based PDT treatments are in clinical trials (Table 9), demonstrating that although there is a promising potential for these systems, there are still significant scientific obstacles that need to be resolved before seeing them in more clinics.

In summary, we would like to emphasize that although PDT and PTT show great therapeutic potential, they also come with adverse effects. For example, in the AuroLase PTT therapy (ClinicalTrials.gov: NCT00848042): 20% (1 participant) presented a cardiac event, 20% (1 patient) presented numbness, and another 20% (1 patient) presented neoplasm at the low and medium treatment levels. Additionally, non-serious side effects like Hyperkalemia (20%), Chills (20%), gastroesophageal reflux (20%), hypertension (40%), sinus tachycardia (20%), flushing (20%), hypoxia (20%), neoplasm related pain (40%), influenza (20%) and others were also observed. Concerning PDT, the use of BF-200 ALA gel (nanoemulsion) (ClinicalTrials.gov: NCT01966120) had shown a great overall patient response (all treated actinic keratosis lesions were cleared) for the 90.9% of patients, compared to the 21.9% of patients when a placebo nanoemulsion was used. Other primary and secondary outcome measures were also set, and more information can be found on the clinicaltrials.gov website. Nevertheless, serious adverse events like acute myocardial infarction (1.82%), femoral neck fracture (1.82%), bursitis (1.82%) and cardiovascular accidents (1.82%), and non-serious adverse events like pain (96.36%), erythema (92.73%), pruritus (38.18%), scab (36.36%), exfoliation (30.91%), oedema (21.82%), vesicles (10.91%), discomfort (9.09%), discharge (5.45%) at the site of application, and nasopharyngitis (10.91%) were also reported. The above numbers suggest that adverse events are unavoidable, and each therapeutic approach should be carefully considered individually and at a patient level before being applied, especially if the treatment affects the patients’ quality of life. Concluding, we want to reiterate that more clinical data are needed before deciding if PDT and PTT are useful or harmful.

## 5 Conclusions and future perspectives

Over the past few decades, the excellent therapeutic effect and great promise of PT has been witnessed and proved in various diseases clinical treatment, including in cancer treatment. To overcome the shortcomings of the light-induced cancer TT in early stage, photosensitive agents-based cancer PT was created and developed. Moreover, as NPs were introduced into cancer therapy and flourished, NPs-based photosensitive agents emerged and have been developed by fast advances due to the unique capacities , i.e., overcoming the deficiencies emerged in photosensitive agents-based PT research and clinical trials. In return, the huge improvement of the cancer PT treatment efficiency further stimulated the explosive development of the NPs-based photosensitive agents. Furthermore, the fabricated NPs can be used as versatile nanosystems for cancer combination therapies. Some NPs have been developed as theranostic tools as well, guiding their cancer PT with the imaging capacity. These progresses demonstrated the necessity, importance and feasibility of the NPs-based PT in cancer treatment.

However, in most cancer clinical treatments, cancer PT still only assumes the auxiliary functions despite its long history. The clinically proved or commercialized NPs-based cancer PT agents are still rare compared with the number of the studies, which could be ascribed to the uncertain penetration and accumulation of the NPs in the deep tumor considering the size of the NPs and its unguaranteed intratumorally cargo release. Other obstacles include NPs’ biosafety concerns to the body after the administration and the limited penetration depth of the light source.

To improve the treatment efficiency and overcome the current obstacles appeared in the current research and clinical trials, we proposed the following four research directions, which should be given more attention for the further investigation.

First, the targeting ability of the NPs for the cancer PT should be improved no matter the NPs are used as the photosensitive agents or the delivery platform. In the case of passive targeting, the morphology parameters of the NPs (*e.g.*, size, shape, or the surface charge) are critical for the delivery efficiency because of the EPR effect requirement. For example, the suitable size and convertible surface charge can help improve the passive targeting of the NPs. The size of the NPs should be less than 250 nm but larger than 10 nm to ensure that they can be delivered to the tumor site without easy renal clearance. As for the surface charge, the ideal design of the NPs surface charge should be negative to alleviate the cell toxicity after the injection, but then after the accumulation in the tumor, if the negative surface can be converted to positive surface charge, it can prolong the retention of the NPs in the tumor. However, if the NPs delivery rely on the active targeting, besides the morphology factors, the tumor-specific marker modified on the NPs becomes a very important factor. The active targeting markers (*e.g.*, antibodies, aptamers, or moieties) have been developed widely and used successfully in the research, but considering the reality of the clinical trials, the tumor anatomical structure of the patients’ needs to be investigated as the first step to provide basis for the next step of the NPs modification. The current ways to ensure the reproducibility of the tumor-specific marker are the overall quantitative methods, such as the fluorescence-quantification or the UV-quantification, but for more reproducible results, the monomer quantification at NPs level needs to be investigated more.

Second, the NPs' responsiveness to TME should be improved. For better cancer PT efficiency, many NPs have been fabricated to be responsive to the complex TME factors, especially for the cancer PDT. Some of them can respond to the slightly acidic environment to shrink the NPs morphology for deeper intratumoral penetration. Some can respond to the excessive chemicals in the tumor (*e.g.*, H_2_O_2_ or GSH) to relieve the hypoxia and the resistance for the chemotherapy. However, the response-sensitivity of the NPs should be enhanced. For example, the pH difference between the normal tissue (pH ∼7.2) and tumor tissue (pH ∼6.8) is small, about 0.3–0.7. In such small range, the requirements for the responsiveness of NPs are stricter, which can be investigated more.

Third, the theranostic ability of the NPs should be improved. The cancer PT started with the light irradiation, which means the light should be irradiated on the tumor tissue when the NPs accumulate most inside the tumor. If the NPs need to release the PSs or have the ability to modulate the TME, the choice of the treatment timepoint needs to be considered more. These needs require the NP-based cancer PT systems preferably to be monitored in real time. With this ability, the light irradiation can be optimized for the better treatment time and treatment-coherence.

Last but not least, the biosafety of the NPs should be investigated more. In most studies, the biocompatibility of the NPs have been confirmed *in vitro* or *in vivo*. But when they need to be transferred to the clinical trials, the longtime biosafety (*e.g.*, biocompatibility, biodegradation) impact research on the human cells and human body are still few, especially when some NPs have been reported that they can possibly induce the cells apoptosis, tissue inflammation, even the DNA replications. Therefore, more biosafety investigations of the NPs-based cancer PT system are needed, such as the phototoxicity of the photosensitive agents before and after the light irradiation, the biodistribution after the injection, or the biodegradation pathway in different organs.

With the in-depth studies on the NPs-based PT agents and the development of the optical fibers and endoscopy, those mentioned concerns have been alleviated and will be solved one day. Although the NPs-based cancer PT is still in its “infant stage”, it has achieved the huge progress in cancer treatment with the development of the NPs. We envision that NPs-based cancer PT will have sustained progress in the future.

## Ethics approval and consent to participate

Not applicable. No clinical study, animal experiments, or human subjects.

## CRediT authorship contribution statement

**Jiachen Li:** Conceptualization, Writing – review & editing. **Shiqi Wang:** Writing – review & editing. **Flavia Fontana:** Writing – review & editing. **Christos Tapeinos:** Writing – review & editing. **Mohammad-Ali Shahbazi:** Writing – review & editing. **Huijie Han:** Writing – review & editing. **Hélder A. Santos:** Conceptualization, Writing – review & editing, Supervision, Funding acquisition.

## Declaration of competing interest

The authors declare no conflict of interest.

## Abbreviations

17AAG: allylamino-17-demethoxygeldanamycin
^1^O_2_: singlet oxygen
2PMI-AQ: two perylene monoimide moieties-diamino anthraquinone
5-ALA: 5-aminolevulinic acid
Ada-BODIPY: adamine-conjugated aza-BODIPY
Ada-PTX: Adamantane-conjugated PTX
Ag: silver
AIE: aggregation-induced emission dyes
ALA-PAMAMs: 5-aminolevulinic acid-polyamidoamine
AlPc: aluminum phthalocyanine
AMPK: 5′ adenosine monophosphate-activated protein kinase
ATO: atovaquone
ATP: adenosine triphosphate
Au: gold
BATA: bis-(alkylthio)alkene
BDP-T-N: 2-ethynylthiophene and 4-(dimethylamino)benzaldehyde modified boron dipyrromethene
Bi_2_Se_3_: bismuth selenide
BODIPY: Boron dipyrromethene amphiphile
BP: beidaphyrin
BPL: beidapholactone
BSA: bovine serum albumin
CAOPLNSs: CaAl_2_O_4_:Eu,Nd nanosheets
CAT: catalase
CdS: cadmium sulfide
Ce6: Chlorin e6
Ce6-Pdots: Ce6-doped semiconducting polymer dots
COFs: covalent organic frameworks
CPPO: bis[2,4,5-trichloro-6-(pentyloxycarbonyl)phenyl] oxalate
CRISPR: Clustered Regularly Interspaced Short Palindromic Repeats
CT: computed tomography
CTPP: 3-carboxypropyl triphenyl-phosphonium bromide
Cu: copper
DBP-Hf: 5,15-di(*p*-benzoato)porphyrin-Hf
DCs: derived dendritic cells
DDSs: drug delivery systems
DHyCUB: hybrid cubosomes loaded with up-converting NPs and daunorubicin
DOX: doxorubicin
DPPC: 1,2-dipalmitoylsn-glycero-3-phosphatidylcholine
DSPE-PEG: 1,2-Distearoyl-sn-glycero-3-phosphoethanolamine-N-[amino(poly(ethyleneglycol))]
DTX: docetaxel
E7: Human papillomavirus oncogenic protein
EPR: enhanced permeability and retention
ER: endoplasmic reticulum
F54: 54 fluorine-19
FA: folic acid
Ftn: ferritin
GNR: Au nanorods
Gox: glucose oxidase
GSH: glutathione
H&E: hematoxylin and eosin
H_2_O_2_: hydrogen peroxide
HA: hyaluronic acid
Hf: hafnium
HIF: hypoxia-inducible factor
HMSNs: hollow mesoporous silica NPs
HPD: haematoporphyrin derivate
HSPs: heat shock proteins
ICG: indocyanine green
IrPVP: iridium(III) complex conjugated with hydrophilic poly(N-vinylpyrrolidone)
LSPR: localized surface plasmonic resonance
MC: Mesoporous carbon
MCN: mesoporous carbon nanoparticle
MeWo: human melanoma granular fibroblasts
MnO_2_: manganese dioxide
Mn_x_O_y_: manganese oxide
MOFs: metal-organic frameworks
MRI: Magnetic Resonance Imaging
MSN: mesoporous silica NPs
mTHPC: metatetra(hydroxyphenyl)chlorin
Nd^3+^: trivalent neodymium
NIR: near-infrared
nMOFs: nanoscale MOFs
NPs: nanoparticles
PA: photoacoustic
PAI: photoacoustic imaging
PARP: poly (ADP-ribose)polymerase
PB: p-phenylboronic ester
PCB: poly(cyclopentadithiophene-alt-benzothiadiazole)
PCMs: phase change materials
PDA: polydopamine
PDT: photodynamic therapy
PEG: polyethylene glycol
PEI: polyethyleneimine
PFH or PFC: perfluorohexane
PFOB: perfluorooctyl bromide
PGL: porphyrin grafted lipids
PHC: PDA-hemoglobin-Ce6
PL: persistent-luminescence
PLGA: poly(lactide-co-glycolide)
PLNPs: persistent-luminescence-based NPs
PM: platelet membranes
PP: OHC-PEG-CHO and PEI
PpIX: protoporphyrin IX
PPy: polypyrrole
PQC NF: PQC-based fiber-forming nanoPSs
PQC: Quinolinium conjugate
PS-g-PEG: amphiphilic poly(styrene-co-chloromethyl styrene)-graft-poly(ethylene glycol)
PSiNPs: porous silicon NPs
PSs: photosensitizers
PT: phototherapy
PT2: polythiophene quaternary ammonium salt
PTT: photothermal therapy
PTX: paclitaxel
PVA: poly vinyl alcohol
QDs: quantum dots
QY: quantum yield
RBCM: red blood cell membrane
ROS: reactive oxygen species
RT: radiotherapy
Ru: Ruthenium
SAE: single atom enzyme
siRNA: small interfering RNA
SOD1: superoxide dismutase 1
SWCNTs: single walled carbon nanotubes
TA: trimethylammonium
TdT: terminal deoxynucleotidyl transferase
TiO_2_: Titanium dioxide
Tm: transition temperature
TME: tumor microenvironment
TOS: α-tocopherol succinate
TPGS: D-α-tocopheryl polyethylene glycol (PEG) 1000 succinate
TPP: triphenylphosphine
TPPC6-OH: 5-(4-(6-hydroxyhexyl) phenyl)-10,15,20-triphenylporphyrin
TPPS_4_: 5,10,15,20-Tetrakis (4-sulfonatophenyl)-porphyrin
TPZ: tirapazamine
TRP: P(AAm-co-AN)
TT: thermal therapy
UCNPs: upconversion NPs
UR-Cyan: RB-encapsulated UCNPs onto cyanobacterial
URG: uninterrupted ROS generator
UV: ultraviolet
VER: verteporfin
ZnPc: zinc phthalocyanine
β-CD: β-cyclodextrin
